# Metacontrast masking does not change with different display technologies: A comparison of CRT and LCD monitors

**DOI:** 10.3758/s13428-024-02526-w

**Published:** 2024-12-30

**Authors:** Tomke Trußner, Thorsten Albrecht, Uwe Mattler

**Affiliations:** Department of Experimental Psychology, Georg Elias Müller Institute, University of Goettingen, Goßlertstr. 14, 37077 Goettingen, Germany

**Keywords:** Visual masking, Consciousness, Display technologies, LCD, CRT

## Abstract

Most vision labs have had to replace the formerly dominant CRT screens with LCDs and several studies have investigated whether changing the display type leads to changes in perceptual phenomena, since fundamental properties of the stimulation, e.g., the transition time between frames, differ between these different display technologies. While many phenomena have proven robust, Kihara et al. (2010) reported different metacontrast masking functions on LCDs compared to CRTs. This difference poses a challenge for the integration of new LCD-based findings with the established knowledge from studies with CRTs and requires theoretical accounts that consider the effects of different display types. However, before further conclusions can be drawn, the basic findings should be secured. Therefore, we tried to reproduce the display type effect by comparing metacontrast masking on an LCD and a CRT in two experiments. Our approach differs from the previous study by increasing the power and reliability of the measurements and carefully matching the two display types. In addition to display type, we varied target-mask stimulus-onset asynchrony (SOA) and stimulus-background polarity. Regardless of display type and polarity, we found the typical type-B masking functions. Evidence for a SOA-dependent display type effect in the black-on-white polarity condition from Experiment 1 was not replicated in Experiment 2. Overall, the results indicate that metacontrast masking effects on objective and subjective measurements, i.e., discriminatory sensitivity and phenomenological reports, do not vary significantly with display technologies. This lack of display effects is discussed in the context of current theories of metacontrast masking.

**Supplementary Information:**

The online version contains supplementary material available at 10.3758/s13428-024-02526-w.

## Introduction

Much of the knowledge about vision is based on decades of research with cathode ray tube (CRT) monitors. However, technological developments are forcing most vision labs to adopt new inexpensive liquid–crystal display (LCD) monitors when the old CRT monitors break down. In light of this change, the question arises whether new findings with LCDs can be seamlessly integrated with established knowledge from research with CRTs. Therefore, a number of studies have investigated the extent to which perceptual phenomena known from research with CRTs can also be found with LCD displays. Interestingly, for many phenomena, no differences have been found when the type of display is changed (see General Discussion). However, metacontrast masking seems to be an exception (Kihara et al., 2010). Therefore, we attempted to replicate the effect of display type on metacontrast masking. In the following, we briefly introduce metacontrast masking before describing the differences between LCD and CRT displays. We then discuss the experiment of Kihara and colleagues (2010) and contrast it with the approach of the present study.

### Metacontrast masking

When a target stimulus is shortly followed by a surrounding masking stimulus at the same location, the visibility of the target can be substantially reduced even though the target and mask do not overlap in space or time—a phenomenon called metacontrast masking (Breitmeyer & Öğmen, 2006). Metacontrast masking has been widely used to investigate the hierarchical structure and temporal aspects of visual processing (e.g., Albrecht & Mattler, 2016; Bachmann, 2007; Breitmeyer & Öğmen, 2006; Francis, 2007). In addition, the rich phenomenology that can be observed with metacontrast masking provides an opportunity to identify and dissect the processes that are involved in the generation of conscious perception (e.g., Albrecht & Mattler, 2016; Koster et al., 2020; Kraut & Albrecht, 2022). Beyond this, metacontrast masking has been used as a blinding technique for studying the processing of unconscious stimuli (Breitmeyer, 2015; Eimer & Schlaghecken, 1998; Fehrer & Raab, 1962; Mattler, 2003; Vorberg et al., 2003).

**Fig. 1:**
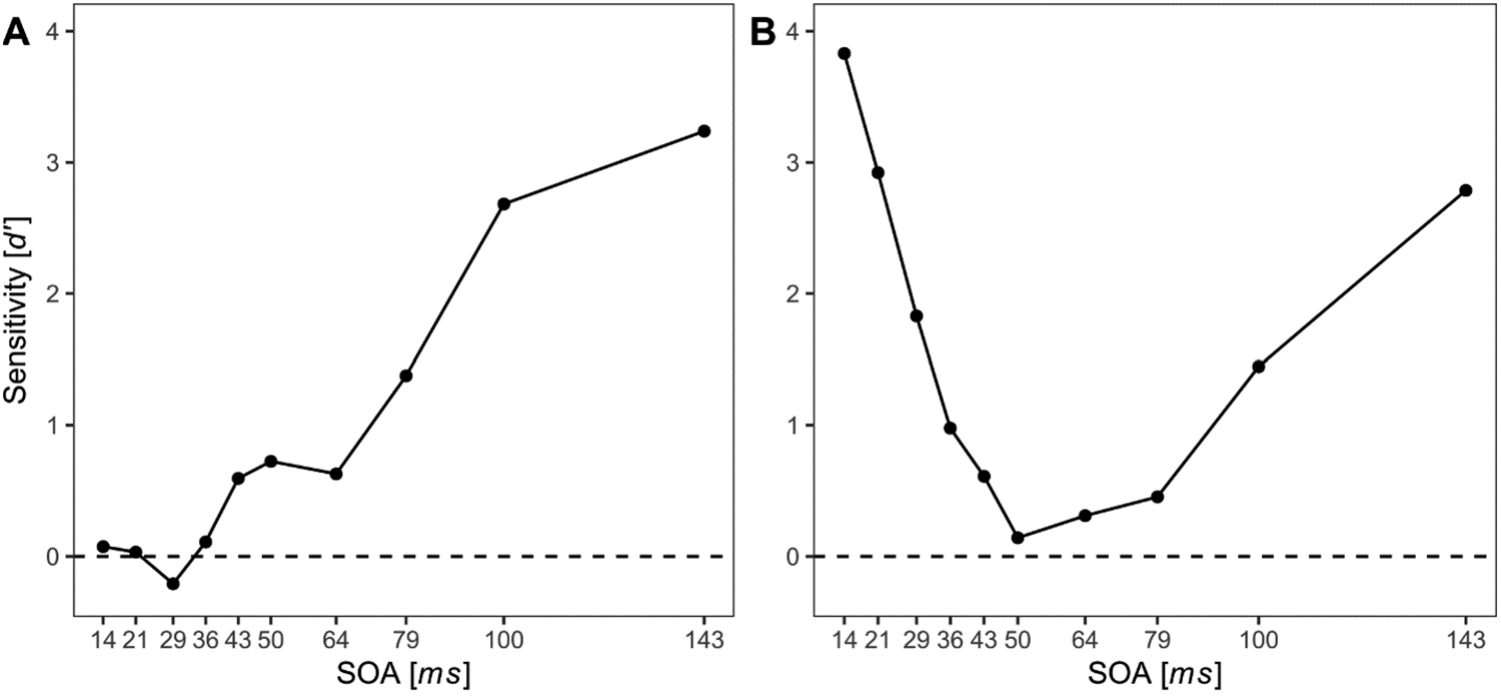
Exemplary metacontrast masking functions of type A (**A**) and type B (**B**). *Note.* Masking functions are shown in terms of measurements of the discriminatory sensitivity *d′* as a function of stimulus onset asynchrony (SOA). The figures are based on unpublished individual data from two different individuals and are shown only to illustrate the descriptions in the text

A crucial feature of the metacontrast masking effect is its dependence on the stimulus onset asynchrony (SOA) between target and mask (Di Lollo et al., 2004; Kahneman, 1967). Changing the SOA by only 10–20 ms can have a substantial impact on target visibility. When masking effects are determined as a function of SOA (often referred to as a masking function), two basic shapes can be distinguished: Type-A functions show strong masking effects at short SOAs and monotonically increasing visibility with longer SOAs. Type-B functions, in contrast, show a U-shaped time course with strong masking effects at intermediate SOAs and high visibility with short and long SOAs (e.g., Kolers, 1962). See Fig. 1 for exemplary individual masking functions of each type. A major part of the research on metacontrast masking is concerned with how other variables affect the masking function and aims to model these relationships and explain their genesis (e.g., Francis, 2007).

A number of variables that influence the masking function have been reported in the literature. Figural characteristics of the stimulus material (Albrecht & Mattler, 2012a; Ansorge et al., 2007; Duangudom, 2007; Francis & Cho, 2008; Koster et al., 2020), retinal locus of stimulus presentation (Bridgeman & Leff, 1979; Saunders, 1977; Zhaoping & Liu, 2022), the criterion contents by which observers decide about their response (Breitmeyer et al., 2006; Kahneman, 1968; Ventura, 1980), light adaptation level (Bischof & Di Lollo, 1995; Purcell et al., 1974), viewing condition (Bruchmann et al., 2016; Schiller & Smith, 1968), and individual characteristics of the observer (Albrecht & Mattler, 2012a, 2012b, 2016) jointly contribute to the shape of the masking function.

Beyond this, however, even inconspicuous details of the stimulation characteristics can affect the masking function, including small changes of the duration (Breitmeyer, 1978a), intensity (Fehrer & Smith, 1962), and spatial separation of the stimuli (Growney et al., 1977). Due to this sensitivity of metacontrast masking to small changes of stimulation characteristics, we thought it worthwhile to examine whether current technological developments play a role in metacontrast masking.

### CRT and LCD devices

A technological development that possibly affects metacontrast masking due to changes in detailed stimulus characteristics is the replacement of cathode ray tube (CRT) by liquid crystal display (LCD) technologies as the prevalent device for stimulus presentations. While basic properties like nominal spatial and temporal resolution or frame-based luminance can be well aligned between these display types, more fundamental differences of physical aspects are inevitable and could influence the outcome of the paradigm under study. Furthermore, an exhaustive investigation of the effects of display technology should also consider their interaction with stimulus–background polarity, since physical differences in stimulation characteristics vary between black stimuli on a white background (B/W) and white stimuli on a black background (W/B). In the following, we will briefly review differences that might affect metacontrast masking. A thorough overview of the underlying working principles of both technologies can be found, for example, in Elze and Tanner (2012).

**Fig. 2:**
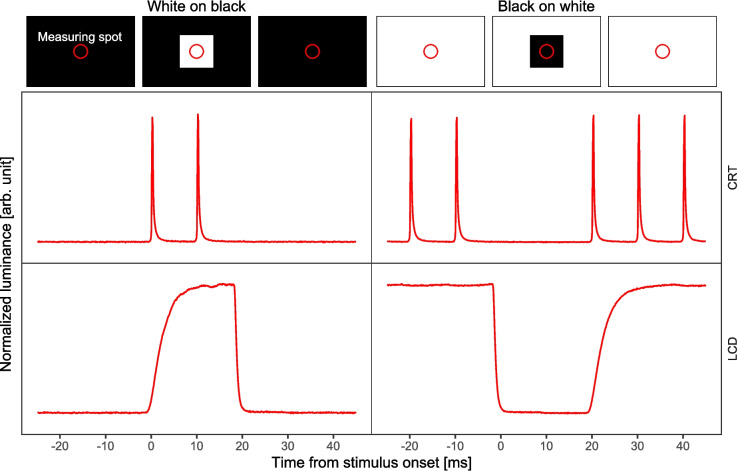
Luminance signal change of a single pixel in a simple stimulus sequence. *Note.* Left column depicts a change from black to white to black; right column depicts a change from white to black to white. First row shows a corresponding stimulus sequence on the screen and the location of the measured pixels (red circle). Second and third rows give recordings of the actually measured luminance of the CRT and LCD devices used in the current study, respectively. Time is arbitrarily locked to stimulus onset. See text for further explanation and Supplementary Material S1 for details on the method for measuring the luminance signal

A major difference between the two technologies concerns temporal aspects of the stimulation. These are best assessed via the time course of the luminance signal emitted by one addressable picture element (pixel) of a display (Fig. 2, top row). For CRTs this signal is pulsed, meaning that each pixel is activated only once per frame (see Fig. 2, second row). The luminance pulse that originates from this activation consists of an almost instantaneous (approximately 0.1 ms) rise to maximum luminance and a slower, exponential decay over the course of roughly 2 ms (Elze, 2010a). In contrast, LCDs are “sample-and-hold” devices. When prompted, the luminance of a pixel starts to ramp up to its chosen intensity and is held static at that intensity until a change is requested (see Fig. 2, third row). Here, the luminance signal rises exponentially and takes considerably longer (about 10 ms or approx. 1 frame at a 100-Hz refresh rate) to reach the maximum of the intensity compared to a CRT. The decrease of luminance, however, proceeds in a very similar way as observed on CRTs over the course of 2–3 ms. Note that decay times on CRTs depend crucially on the built-in phosphor type and can vary substantially (Di Lollo et al., 1997). Transition durations of LCDs also depend on constructional aspects of the model (Lagroix et al., 2012) and are further impacted by the chosen start and end point of an intensity change (Elze & Tanner, 2012). Therefore, the durations given above are valid only for full transitions between black and white on the specific display models used in this study.

Two aspects that are relevant for metacontrast masking are influenced by the technological differences described above. First, the pulsed and static characteristics of the luminance signal produce stimuli with fundamentally different physical properties, and this could have consequences for their processing. Rohr and Wagner (2020) assumed that, in accordance with Bloch’s law (Gorea, 2015), no effects should be expected if the total energy within a certain time interval stays the same. We do not think that this is necessarily so in the case of metacontrast masking, since the role of integrated stimulus energy in metacontrast masking, typically assessed through the ratio of target and mask stimulus energy (M/T energy ratio) is known to be complex. As a broad rule, M/T energy ratios ≤ 1 tend to produce type-B while ratios > 1 tend to result in type-A masking functions (Weisstein, 1972). How sensitive the masking function can be to this factor has been demonstrated by Breitmeyer (1978a). By doubling the M/T energy ratio from 0.5 to 1—in this case a difference of only 8 ms in mask duration—a type-B masking function was almost completely converted into a type-A masking function. Matters are further complicated by the fact that equal M/T energy ratios, stemming from different combinations of stimulus durations and intensities, can lead to different masking functions (Breitmeyer & Öğmen, 2006). The simple M/T energy ratio estimated by Bloch’s law therefore does not suffice to fully explain all aspects of metacontrast masking functions. As argued by Breitmeyer and Öğmen (2006, pp. 49–50), Bloch’s law might only be an appropriate model for stimulus energy up to a critical integration time interval. It is unclear how long this critical interval exactly lasts, especially since different visual processes could in principle have different integration characteristics.

Beyond this, the literature reports effects of luminance flicker with rates of up to 100 Hz at visual processing levels up to V1 in conditions where the stimuli themselves were not perceived as flickering (Gawne & Woods, 2003; Herbst et al., 2013; Krolak-Salmon et al., 2003; Williams, 2004; Zele & Vingrys, 2005). For instance, Williams and colleagues (2004) found neurons in monkey’s V1 which respond to flicker rates as high as 100 Hz when high-contrast patterns are used. In presurgical epileptic patients, Krolak-Salmon et al. (2003) recorded steady-state visual-evoked potentials responding to CRT video-screen flicker in the lateral geniculate nucleus (LGN), optic radiation, and the V1/V2 complex (see also Herrmann, 2001). Hence, Williams et al. (2004) inferred that “whenever a person is watching television on a CRT monitor, his/her V1 cortex is being buzzed at the video refresh rate” (p. 8288). These findings suggest that early visual processes respond differently to stimuli on the pulsed CRT display than to stimuli on the static LCD. Additionally, when identical frame-based luminance levels between display types are desired, it is important to note that the maximum intensity of a single CRT peak must be considerably higher than the intensity of the static LCD signal (see below). To our knowledge, the extent to which the resulting differences in the details of the stimulation affect processes that contribute to metacontrast masking is currently unclear.

The pulsed and static characteristics of the luminance signals of CRT and LCD, respectively, lead to another difference between display types when considering different contrast polarities (i.e., white-on-black stimuli vs. black-on-white stimuli). Figure 2 (left column, second row) shows for a CRT that the stimulus material itself introduces relatively small amounts of flicker when a white stimulus is presented on a black background. In the case of a white background, however, it is the background that induces relatively large amounts of flicker into the system (Fig. 2, right column, second row). In the case of LCDs, there is no comparable difference between the two stimulus-background polarities (see Fig. 2, third row). Hence, it is important to distinguish both polarities when investigating whether different display technologies lead to different metacontrast masking functions.

A second aspect that is affected by the dissimilar time courses of the luminance signals regards the transition between successive stimuli. As already described by Elze (2010b), the slowly rising luminance signal on LCDs can produce artifacts in the transition from previously presented stimuli to the following frame. In the case of metacontrast masking, the effective stimulus durations and the effective interstimulus intervals (ISI) between target and mask might differ from the durations and ISIs that are requested and specified in the computer program that controls stimulus presentation. Again, this matter is further complicated by the stimulus-background polarity, because the time courses of the rising and the falling luminance signals are not symmetric, at least on the LCD used in the present study.

**Fig. 3:**
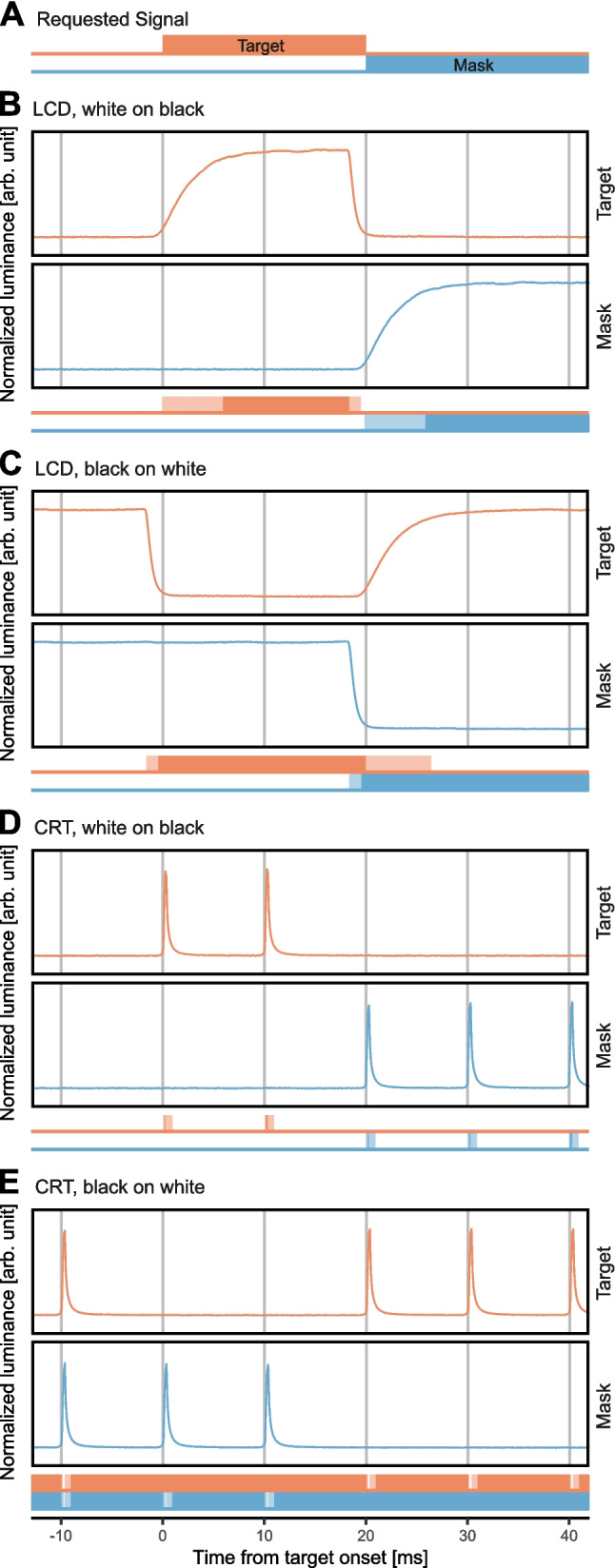
Requested and actual signal changes for LCD and CRT displays. *Note.* **A** Requested stimulus onsets and offsets for a target presented for 20 ms (orange) followed by a mask with a SOA of 20 ms and a duration of 120 ms (blue). Note that the offset of the mask (duration of 120 ms) lies outside the displayed time interval. **B**–**E** Normalized luminance signals for the target-mask sequence in (**A**) as measured at the screen surface (orange and blue lines in the top panel, respectively) for all four display type × polarity conditions. The colored ribbons below each panel depict the polarity-dependent signal threshold state of the respective pixel at a given time. Thresholds were based on the ISO 9241–305 standard for response times. Full colored areas represent phases of super-threshold stimulus signal; white areas represent phases of supra-threshold stimulus signal, and pastel-colored areas represent transitional phases. Note that in the condition black on white (**C** and **E**), stimuli are ON when the luminance is low. Also note that for black on white stimuli on a CRT (**E**), considerations about the (non-displayed) background signal are crucial for interpretations about stimulus visibility (see text). Time is locked to target onset. Gray vertical lines mark frame boundaries of 10 ms. See text for further explanation and the method section and Supplementary Material S2 for details on the measurements

To examine this matter in more detail, we measured the target and mask luminance signals across the four combinations of display type and stimulus polarity with an optical transient receiver (DM&S model OTR-3; see method section and Supplementary Material S2 for detailed measurement methods). According to the ISO 9241–305 standard, the response time of a display is defined as the time it takes to change the luminance of a pixel from 90 to 10% of the maximum luminance and vice versa. Following this norm, we consider a white-on-black stimulus to be fully present or absent when the luminance signal exceeds the 90% threshold or falls below the 10% threshold, respectively. For black-on-white stimuli the thresholds are reversed: Whenever the luminance signal lies between 10 and 90%, the stimulus is in a transition phase (depicted in Fig. 3 by pastel colors).^1^

Figure 3 depicts the requested on and offsets for target and mask with an ISI of 0 ms. The following panels show the measured time course of luminance signals for each display type and polarity condition. At first glance, the LCD luminance signal exhibits the expected continuous course (Fig. 3B and C). For white-on-black stimuli (Fig. 3B), the target signal (orange) rises, however, relatively slowly at onset and drops rapidly a few milliseconds before the requested offsets so that the effective target duration is slightly below the requested stimulus duration. Due to the slow rise of the subsequent mask signal, the mask reaches full presence 6.5 ms after the target signal has fallen below the 10% threshold, and thus introduces an ISI that is effectively larger than the requested 0 ms because both signals are below 10% luminance for 0.5 ms. This actually realized ISI is represented in Fig. 3B by the small gap between the end of the pastel orange ribbon and the beginning of the pastel blue ribbon.

The reverse pattern occurs with black-on-white stimuli (Fig. 3C). Due to the steep rise of the stimulus signal and the slow fall, the target duration is slightly longer than requested, and the effective ISI between target and mask is shorter than requested due to the temporal overlap between target and mask. This overlap is at least 0.4 ms when the time between the end of the fully present target and the fully present mask is considered (represented as the overlap between the full-orange ribbon and the full-blue ribbon in Fig. 3C), but it is 7.7 ms when the stimuli are considered including their transition phases (represented by the overlap between the beginning of the pastel blue ribbon and the end of the pastel orange ribbon in Fig. 3C).

By contrast, the CRT produces luminance pulses that remain only 0.8 ms above the 10% threshold, which is considerably shorter than one frame at 100 Hz (Fig. 3D and E). A requested ISI of 0 ms therefore actually incorporates an inter-pulse period of approximately 9 ms, where no stimulus is presented above the 10% threshold. Crucially, this gap is not specific for the case of stimulus transition and it is independent from polarity conditions, because it is a consequence of the inherently pulsed nature of every frame presented on a CRT. Beyond this, it has to be further considered that this feature of CRTs affects not only the stimulus, but also the presentation of the background. When presenting white-on-black stimuli, the background is static like on an LCD, since there is no need for luminance pulses to build up the background. Accordingly, the interpretation of the stimulus signal is straightforward (see Fig. 3D). The last luminance pulse of the target occurs about 10 ms after the requested target onset. With the requested ISI of 0 ms, there is no luminance signal for about 9 ms until the first luminance pulse of the mask is presented, which follows about 20 ms after the requested onset of the target.

On the contrary, when presenting black-on-white stimuli (see Fig. 3E), the white background has to be built up by the pulsed luminance signal. Consequently, a black stimulus is determined by the absence of such a short pulse at the stimulus location during the presentation of the pulses of the surrounding background. The fact that the target signal continuously stays below the 10% threshold from around 9 ms before until 20 ms after the requested target onset does therefore not imply a continuous presentation of the target on the screen, because the target only becomes apparent at the beginning of each frame when the white background is flashed around it. Therefore, the stimulus transition basically follows the same pattern as for the white-on-black polarity condition. To sum up, display type and polarity modulate quite fundamental temporal aspects of the stimulation in terms of the transition times. We think it is plausible that these differences influence metacontrast masking since masking functions are sensitive to temporal parameters of the display in terms of SOA and energy ratio. However, it is currently unclear whether metacontrast masking functions are sensitive to these differences in transition times.

In addition to the temporal differences of the luminance signals, a third source of potential display type effects arises from differences in the details of the spatial resolution between LCDs and CRTs. A well-known problem of CRTs is luminance interactions between nearby pixels (Pelli, 1997) and veiling glare, which is the diffuse spreading or scattering of light within various parts of a display (Krupinski et al., 2004). This leads to blur and a reduction of the actual resolution compared to the resolution specified by the nominal pixel size. Since these problems do not occur with LCDs, their actual resolution approaches the specified pixel size (Saunders & Samei, 2006). For small, fine-grained stimulus materials, the blurred CRT image presentation potentially changes the luminance profile of critical areas of the stimulus and may thereby alter contour proximity, which is also related to the spatial gap between the target and mask in the case of metacontrast (cf. Kihara et al., 2010). Small variations of this gap can have substantial impact on metacontrast masking functions. When the gap between the target and mask increases from 1° to 10° of visual angle, Growney et al. (1977) found a reduction of masking strength by almost half. Therefore, it seems reasonable that the differences in effective resolution between display types might lead to stronger masking on CRTs than on LCDs. Knowledge of such influences would be important information to consider when studies attempt to replicate findings made with a CRT using an LCD. A practical significance of such findings arises from the fact that some contour effects could be compensated by appropriate changes in the stimulation parameters on the LCD.

### Evidence for effects of display type

Before we approach the question of the influence of display technology on metacontrast masking, we would like to provide a brief overview of experimental studies on the effect of display technology on other visual phenomena. To anticipate: The findings are mixed and suggest that a simple general prediction of effects seems to be difficult.

Kihara and colleagues (2010) examined the attentional blink effect and employed white stimuli on a black background. The attentional blink effect occurs when two target stimuli follow each other in a rapid stream of distractors (Raymond et al., 1992). Detection performance of the second target stimulus is reduced when it is preceded by the other target stimulus within approximately 300 ms. Kihara and colleagues found typical attentional blink effects with both LCD and CRT display types, but these effects were modulated by display type because the detection rate of the second but not the first target was lower on LCD. According to Kihara and colleagues, this effect could be caused by the sharper edges of the stimuli on the LCD which may have enhanced masking effects of the distractors.

Klein et al. (2013) compared contrast sensitivity for both static and flickering gratings on CRT and LCD devices and found no differences in a discrimination task. Hollands et al. (2002) compared performance in a visual search task with different stimulus colors (red, blue, and white) between CRT and LCD and found no differences in detection performance or response time as well, at least when the viewing angle of displays was properly controlled. Bognár et al. (2016) reported equal discrimination performances for the double flash and the flicker illusions for both display types. Stein et al. (2020) replicated the motion bridging effect, which is an illusory motion that integrates the directional signal of a subliminal fast rotation, with an LCD as previously found with a CRT oscilloscope.

Rohr and Wagner (2020) compared priming effects and prime visibility in a masked-pattern number-priming task and reported no fundamental differences between display types. Exploratory analyses of Rohr and Wagner (2020), however, revealed a significant three-way interaction between display type, prime congruency, and notation match between prime and target numbers (Arabic numerals vs. Latin script): Priming effects were reduced on the LCD when the prime and target notation matched. Kunde et al. (2003) reported a similar two-way interaction in a study, however, where stimuli were shown on a CRT, a condition where Rohr and Wagner did not find the interaction. The average priming effects reported by Kunde et al. (8–17 ms) were considerably larger than those reported by Rohr and Wagner (LCD: − 1–6 ms; CRT: 0–4 ms) most likely due to Kunde et al. having a larger target duration, which can reduce the suppressive effects of the forward masks (Becker & Mattler, 2019) and a larger SOA, which increases priming effects (e.g., Vorberg et al., 2003; Wernicke & Mattler, 2019). Therefore, it seems questionable whether this incidentally found interaction has to be considered as evidence for a systematical effect due to CRT and LCD technologies rather than a consequence of low statistical power for this interaction, especially since there was no follow-up experiment run by Rohr and Wagner that has replicated this effect.

One case of unambiguous effects of display type was reported by Lagroix et al. (2012). When a white stimulus was presented on a black background, the authors found considerable amounts of visual persistence on a CRT device (up to 125 ms when light-adapted and up to 4 s when dark-adapted), but no signs of visual persistence at all on an LCD. For black stimuli presented on a white background, no visual persistence was observed for either display technology. Interestingly, the authors can rule out an obvious explanation through differences in the falling time of the luminance signal between displays, and a phenomenological report of the authors indicates that the actually presented stimuli had never been visible on screen in any condition. The display characteristics that may have caused the visible persistence on the CRT to be that high has yet to be determined.

### Display type effects on metacontrast masking by Kihara et al. (2010)

In addition to the aforementioned study of the effect of display type on the attentional blink, Kihara and colleagues (2010) reported a first experiment on the effects of display type on metacontrast masking. Again, the authors employed white stimuli on a black background. The merit of the study by Kihara et al. (2010) is that it showed for the first time that typical metacontrast masking effects can be achieved with an LCD, although the details of the masking functions differed between display types. Metacontrast masking was found to be significantly reduced on LCD compared to CRT for the intermediate SOAs of 33 and 50 ms. Although these differences might appear relatively small, they are important for current research on metacontrast, which aims to determine the mechanisms that underlay metacontrast masking. Beyond this, these findings call into question whether future research with LCDs can be seamlessly coalesced with previous findings from CRTs. Kihara and colleagues argued that larger masking effects on CRT might result from the fact that the pulsed luminance signal of the CRT reached higher peak intensities than the signals of the LCD when the frame-based luminance levels were matched between the CRT and the LCD (see above). As an alternative source of the observed effects, the authors discussed the generally increased blurriness of the stimuli on the CRT relative to the LCD. According to this view, the blur on the CRT might have enhanced the contour proximity between target and mask, which could have increased masking effects. Beyond this, however, the different masking functions in CRT and LCD displays could also indicate much more basic effects arising from the fundamental differences between the two display types mentioned above. In addition, these display type effects would also pose a challenge to current metacontrast masking theories that do not account for display type effects. However, before drawing any further conclusions, the basic finding should be replicated, and the influence of obvious factors that might have caused the differences in metacontrast masking reported by Kihara et al. (2010) should be ruled out.

### The present study

Previous research from our lab indicates significant levels of perceptual learning and interindividual differences in metacontrast masking functions (e.g., Albrecht & Mattler, 2012a, 2012b, 2016; Albrecht et al., 2010). When these individual data are aggregated across a sample, the interindividual differences can lead to misleading effects, especially when the aggregated masking functions come from measurements from consecutive phases of the experiment, as was the case in the study by Kihara and colleagues. In addition, differences in the masking functions could also be due to small differences between the settings of the two displays. Lastly, the reliability of the results of Kihara et al. can be questioned, as the results are based on the data of only 12 participants who participated in a single fairly long session with a total of 1440 trials and only 20 trials per condition. The power of this study was not reported. We think it is important to replicate the display type effects reported by Kihara et al. (2010) because these results suggest that research on metacontrast with LCDs cannot be seamlessly integrated with the previous reports on metacontrast masking found with CRT devices. Furthermore, we think it worthwhile to further investigate the effects of display type, since such effects could prove instructive for understanding the mechanisms underlying the phenomenon by examining the sensitivity of metacontrast masking to the differences in stimulus properties of display types. In the present study, we therefore examined the effects of display type on metacontrast masking with carefully matched settings between the two displays and a more sophisticated paradigm in larger samples of participants who were examined with the two display types on four separate days.

Our display setup consisted of a CRT model (ViewSonic G90fB) which has been frequently used in vision science in the past and a modern-generation LCD display (Dell AW2518HF). Although the LCD that was used by Kihara et al. (2010) is a decade older than the LCD that we used here, the technical specifications given by the manufacturers match reasonably well. The spatial resolution of the two LCDs was comparable, with 38 ppcm (Kihara et al.) and 35 ppcm (in the present study). The average response times were also similar with 5 ms (Kihara et al.) and 4 ms (present study). While Kihara et al. used a 60 Hz refresh rate, we used a 100 Hz refresh rate as recommended for CRT devices by Zele and Vingrys (2005). Kihara and colleagues reported that the luminance of the stimuli were matched to 50 cd/m^2^, without giving detailed information on the resulting background luminance (they only reported background luminance < 0.3 cd/m^2^). In contrast, we matched the luminance between display types as closely as possible (see below) because LCDs tend to be more luminous than CRTs by default due to backlight artifacts. In addition to this, we also explicitly controlled the visible display area of the two displays and the ambient luminance of the lab room.

Beyond this, the present study differs from the study of Kihara and colleagues in a number of points regarding the metacontrast task that was employed. First, the shape of the stimuli differed. Kihara et al. presented diamond-shaped targets with a missing upper or lower corner and a uniform mask with a full diamond cutout. We used diamond- and square-shaped target stimuli and corresponding masks with a star-shaped cutout (see Fig. 5). These stimuli are known to produce high interindividual variability in masking functions and a rich phenomenology (Albrecht & Mattler, 2012b, 2016; Koster et al., 2020). This allowed the investigation of possible effects of the type of display on participants’ phenomenological experiences, which were measured by a questionnaire given to the participants after the experimental sessions. Second, while Kihara et al. used more atypical green stimuli on a black background, we employed more commonly used black and white stimuli and backgrounds. Third, we systematically varied the polarity of the stimuli and the background in this way to investigate whether the abovementioned different characteristics of the two types of displays occurring in the two polarity conditions affect the masking function. Fourth, our study was designed to achieve a higher power than the experiment by Kihara and colleagues by testing two samples with 16 and 24 participants with 104 trials per condition and participant in Experiment 1 and 2, respectively. Finally, to further increase the reliability of our measurements, we gave participants more training on the task by examining them over multiple days in sessions with only 728 trials per session and sufficient rest between trial blocks, rather than in a single large session like Kihara and colleagues. Whereas Kihara et al. changed display types within a single session, we changed display types between sessions, and our participants did not change displays within the same session.

To anticipate our results: Overall, the display type had no significant effect on masking functions in the first experiment. Nevertheless, there was an indication for a modulatory effect of display type in the case of black-on-white stimuli. Therefore, we replicated this condition in Experiment 2, which however did not confirm the indication. A final overall analysis of the pooled data from both experiments across our total of 40 participants revealed no evidence for an effect of display type on metacontrast masking functions.

## Display configuration

As mentioned above, metacontrast masking is sensitive to even small variations in stimulus characteristics. Therefore, it was an important precondition for our experiments to match all display characteristics that can be as closely aligned as possible. In the following section we describe our displays, measurement instruments, and the final display settings. More detailed descriptions of the exact procedures used to achieve a close match can be found in the Supplementary Materials as noted in the respective sections.

### Apparatus

**Fig. 4:**
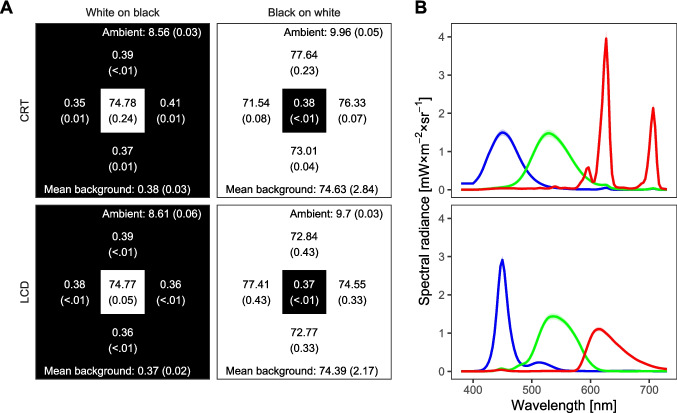
Illustration of the luminance and spectral profiles of the two displays. *Note.* **A** Luminance of black and white areas in cd/m^2^ for CRT (upper row) and LCD (lower row) for two polarity conditions: white stimuli on black background (left) and for black stimuli on white background (right). Each of the four boxes represents the respective visible display area and image configuration. Values within and around the stimulus patch represent the mean of 10 measurements at the respective location with standard deviation given in brackets. Overall means of the background color are given in the lower left corner of each display. Ambient luminance measured from distance of 1 m are given in the upper right corner of each display. **B** Spectral profiles of CRT (upper panel) and LCD (lower panel). Lines represent averages across 10 measurements, with standard deviations represented by a lighter shading of the same color

We used a 19″ ViewSonic G90fB CRT and a 25″ Dell Alienware AW2518HF LCD for stimulus presentation. Both displays were set up on the same table and could be switched between sessions by the experimenter. To match the size of the visible display area, an opaque cover with a 20 $\times$ 20 cm cutout was placed in front of the screen. Furthermore, to achieve consistent ambient luminance of approximately 10 cd/m^2^ (see Fig. 4A) across different experimental conditions, the room was lit by a neon tube attached to the upper back of this cover. Luminance was measured using a Photo Research Litemate PR 524 luminance meter equipped with a contact probe for stimulus measurements and a diffusor for ambient luminance measurements. Spectral radiance profiles and standardized color values (CIELAB color space) were measured using an X-Rite i1 Studio spectrometer at a resolution of 3.3 nm and Argyll Color Management System (CMS) Software (version 2.1.2; Gill, 2020). The luminance signal of the displays was measured with an optical transient recorder (DM&S model OTR-3). The OTR-3 is a commercially available device to measure temporal variations of light intensities which basically consists of a photodiode and an analog-to-digital converter (see Supplementary Material S1 for details on the measurements).

### Matching of spatial characteristics, luminance, and color

To match the size of the pixels between the two displays, the CRT was set to a display resolution of 1024 $\times$ 768 px and the LCD to a resolution of 1920 $\times$ 1080 px. Additionally, the screen area of the CRT was shrunk using the internal width and height controls so that both displays operated with a pixel density of approximately 35 ppcm.

The luminance and color of white and black pixel areas and the resulting ambient luminance were matched as closely as possible between displays and polarity conditions. A detailed description of this matching procedure can be found in Supplementary Material S1. Figure 4 shows the resulting luminance and spectral radiance after matching both displays. Average luminance values ranged from 71.5 to 77.64 cd/m^2^ for white and 0.35 to 0.41 cd/m^2^ for black, and showed little variance between either displays or positions (see Fig. 4A). Ambient luminance ranged between 8.61 and 9.96 cd/m^2^ and was higher when the background was white. Spectral radiance profiles shown in Fig. 4B reveal substantial differences between displays. In particular, the profiles of the blue and the red channels differed between the displays. These differences could not be further reduced by further adjustments.

To avoid differences between the two displays that can arise from faint color tendencies in white or black regions on the screen, we matched the color of the two displays corresponding to the procedure described in the supplements. Table 1 shows the results of the color-matching procedure that we established to achieve a high color similarity between the displays for the two polarity conditions of our study. We use CIELAB color values because RGB values do not provide information about the actual spectral characteristics of the colors.

**Table 1:**
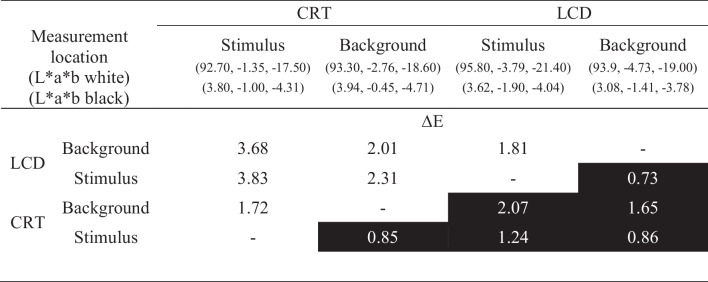
Color adjustments for the two displays in terms of CIELAB and ∆*E* values at different locations of measurements

CIELAB color values are given below in the respective column label (first white, then black). Values represent the average of 10 measurements of each stimulus and 40 measurements of each background. ∆*E* values are measures of color differences for comparison between white (upper triangle) and black (lower triangle) parts of the screen. Higher ∆*E* values represent larger differences between perceived colors with 1 defined as the just noticeable difference

On average, a white pixel had values for L*a*b of 93.9, − 3.16, and − 19.10, and a black pixel had values for L*a*b of 3.61, − 1.19, and − 4.21. Differences in color were assessed through ∆*E* values (Sharma et al., 2005). ∆*E* = 1 is defined as a just noticeable difference (Mandic et al., 2006), and higher values indicate larger color differences. In our setup, we measured a maximal difference of ∆*E* = 93.3 between black and white. Compared to this range, our final match was reasonably close for all comparisons, with black image parts showing an overall better match than white parts. The most pronounced differences remained between the white stimulus on the CRT and the white LCD stimulus (∆*E* = 3.83), and between the white CRT stimulus and the white LCD background (∆*E* = 3.68). However, it should be noted that ∆E values > 1 occurred even within the same display. Considering the inevitable differences of the spectral profiles, the relevance of the remaining Δ*E* differences for the masking paradigm appears questionable to us. From a subjective point of view, the colors seemed to match reasonably well. This is corroborated by participants’ reports in the debriefing, where only 8 out of 40 participants answered the question of whether they noticed some color difference between the displays.

## Experiment 1

### Methods

#### Participants

A total of 17 participants (14 females, ages between 19 and 30 years, *M* = 21.56 *SD* = 2.92) took part in our study. One participant refused to continue after the first session and was excluded from data analysis. None of the participants had participated in a study on metacontrast masking before, and all were naive to the precise background of the study. All had normal or corrected-to-normal vision and reported no history of mental or neurological disorder. All participants gave informed written consent before attending the experiment and received either a monetary reward or course credit. All experiments were approved by the local ethics committee of the Georg-Elias-Müller-Institute of Psychology, University of Göttingen, and all experimental procedures are in accordance with the Declaration of Helsinki.

#### Sample size rationale

In multifactorial within-subject designs, it is not trivial to determine a priori the power and the sample size required for a particular effect, because it depends on many usually unknown factors, such as the number of trials per condition, the measurement precision of individual measurements, the correlations between the repeated measurements, and the individual differences in the effects (e.g., Baker et al., 2021; Biafora & Schmidt, 2020; Brysbaert & Stevens, 2018). Interindividual differences, which often occur with meta-contrast masking (Albrecht & Mattler, 2012a, 2016; Albrecht et al., 2010; Maksimov et al., 2011), can have a strong influence on correlations between repeated measurements and thus are a crucial factor in determining the statistical power of the test.

We conducted simulations to estimate the expected statistical power for different sample sizes and different true effect sizes as follows: First, to account for the typical individual differences that were found in several previous studies using the same stimuli as in the present study (Albrecht & Mattler, 2012a, 2012b, 2016; Albrecht et al., 2010), we calculated the variance–covariance matrix from empirical data from studies of our own lab comprising the same stimuli and SOA conditions. Second, we based our assumptions about the true effect on the data reported by Kihara et al. (2010). However, our design differs in the number of SOAs and display types. Therefore, we could not transfer the effect size ${\eta }_{P}^{2}$ to our design. Instead, we calculated the differences between the empirical means in Kihara et al. (2010) in each of the seven SOAs that matched our own SOAs as closely as possible and used this as the basis for estimating the “true” means. For each of three sample sizes (*n* = 12, 14, 16) we simulated 10,000 data sets drawn from a multivariate normal distribution with the estimated “true” means and the estimated variance–covariance matrix and conducted repeated-measures analyses of variance (ANOVAs) to test for a main effect of display type and for an interaction of display type × SOA using Greenhouse–Geisser-corrected degrees of freedom.

Under the assumption of a true effect of the same magnitude as the empirical effects in Kihara et al. (2010), a sample size of *n* = 16 participants yielded a statistical power of > 99% for the main effect of display type (${\eta }_{G}^{2}$ = 0.05) and a power of 85% for the interaction of display type × SOA (${\eta }_{G}^{2}$ = 0.01). To assess the minimal difference between display types that we would be able to find, we conducted additional simulations with successively reduced differences between estimated “true means.” For the main effect, the power was still at 81% with an effect size of ${\eta }_{G}^{2}$ < 0.01 (corresponding to a difference for means of only 43% of the empirical effect of Kihara et al.). For such an effect size, a coarse estimation of the power in the original Kihara et al. study yielded a power of only 66%. Note, however, that this estimate did not take into account the lower number of trials per condition in the original study, so the power difference between studies was probably even larger.

As mentioned above, another crucial aspect for statistical power is the precision of individual measurements and therefore the number of trials per participant and condition. Recently, Brysbaert and Stevens (2018) recommended at least 1600 trials per condition across all participants for studies using linguistic stimulus material. With a sample size of *n* = 16 participants and *r* = 104 trials per participant and condition, we adhere to this recommendation (*N* = 1664 trials).

Beyond this, we can calculate the precision at the individual level according to the formula $s/\sqrt{r}$ with standard deviation *s* and number of trials* r* (Eisenhart, 1969). With a binomially distributed response variable (correct/incorrect), the maximum *s* = 0.5 (for 50% correct responses) and decreases to *s* = 0.3 for 90% correct responses. Therefore, the precision at the individual level is between 4.9% (for 50% correct responses) and 2.9% for excellent performance, which exceeds the precision in the original study of Kihara et al. (11.2–6.7%).

#### Stimuli and task

**Fig. 5:**
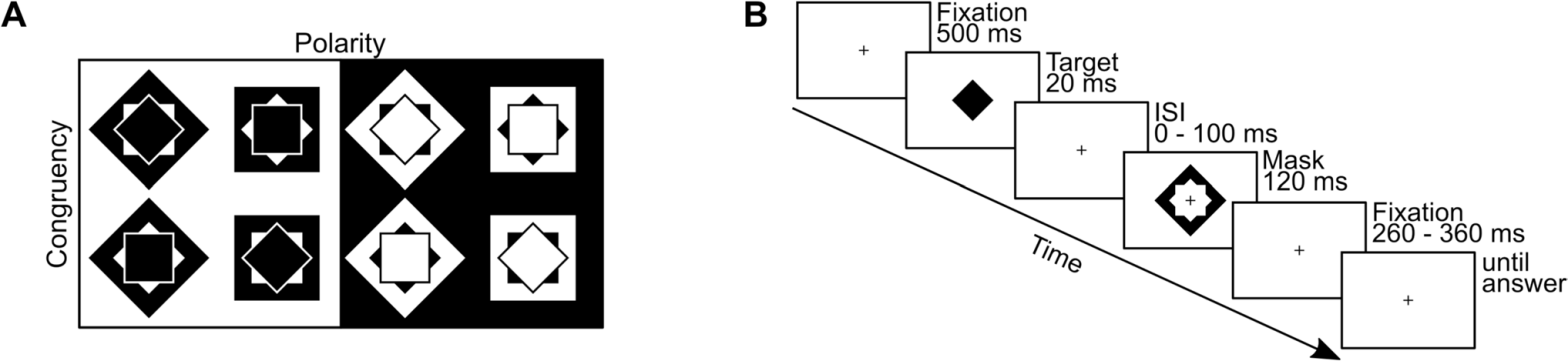
Stimuli and trial sequence of the experiment. *Note.* **A** Congruent (upper row) and incongruent (lower row) combinations of target and mask stimuli for the two polarity conditions black stimuli on a white background (left) and white stimuli on black background (right). **B** Sequence of events in a single trial in the condition with black stimuli on a white background. Note that the computer recorded a response only during the last interval titled “until answer”

Stimuli were adapted from previous studies (Albrecht et al., 2010; Albrecht & Mattler, 2010, 2012b, 2016; Koster et al., 2020; see Fig. 5). A small cross with a diameter of 0.21° of visual angle served as a fixation point and was presented in the center of the screen throughout the trial. Target stimuli were filled squares and diamonds with a diameter of 1.5° of visual angle. Mask stimuli were larger squares and diamonds with a diameter of 2.6° of visual angle and a star-shaped cutout, leaving a gap of 0.02° of visual angle between the inner contour of the mask and the outer contour of the target. Combinations of target and mask could therefore be either congruent (both square or both diamond) or incongruent (one square and one diamond; see Fig. 5A). All stimuli were presented in the center of the screen either in black color on a white background (B/W) or in white color on a black background (W/B). The luminance of white pixels was on average 74.5 cd/m^2^ and of black pixel 0.37 cd/m^2^ (see display configuration above).

Figure 5B illustrates the sequence of events of a single trial. At the beginning of the trial, the fixation was presented for 500 ms, followed by the target stimulus for 20 ms, a variable interstimulus interval (ISI), and the mask for 120 ms. The SOA between the two stimuli was varied in seven steps (20, 30, 40, 50, 60, 80, 100, and 120 ms). Subsequently, but not before 1000 ms after the beginning of the trial, participants could give their response via button press.

Participants were instructed to report the target shape (square or diamond) by pressing either the left or right STRG button, respectively. There was no time pressure, and participants always had to select one of the two alternatives. Following this response, a blank screen was presented for a varying intertrial interval (ITI) of 500 to 1000 ms duration.

#### Design and procedure

We implemented a 2 (display type) × 2 (polarity) × 2 (congruency) × 7 (SOA) within-subject design. For all analyses, the factor congruency was omitted because including congruency as a factor could lead to substantial artificial effects due to response biases (Albrecht & Mattler, 2012a; Vorberg et al., 2003). Thus, the analysis design was a 2 × 2 × 7 within-subject design. Each participant completed a total of 2912 trials (104 trials in each of the 28 conditions for analysis) split across four sessions of 728 trials each. Display type and polarity were varied session-wise, with order balanced across participants using a standard Latin square design. We created four different sequences of our four conditions A, B, C, and D: ABCD, BCDA, CDAB, and DABC. These four sequences were balanced across participants, so that four participants each completed the experiment in the same order. Unfortunately, however, this Latin square design does not allow us to control for all carry-over effects, because we cannot separate learning effects across sessions from interindividual differences between the conditions. At the beginning of the first session, participants were informed about the general purpose of the experiment. Basic demographic information was collected, and the metacontrast task was thoroughly instructed. After completing eight test trials with increased stimulus durations, participants started working through the proper experimental trials. The instructions and test trials were repeated at the beginning of each of the remaining three sessions. At the end of each session, a short questionnaire about the task was completed. Questions were asked by the experimenter and answered verbally by the participant. Aside from questions regarding participant’s condition, performance, and potential strategies, the questionnaire included items regarding their phenomenological experience of the percepts *no target*, *target inside mask*, *target before mask*, *rotation*, and *expansion*, which have been studied by Koster et al. (2020). The percepts *dark target* and *bright target* were merged into an overarching percept called *afterimages*. In addition, we asked for the percept *changes in perceived target duration*. Each percept was described, and participants were asked whether or not they experienced the respective percept. At the end of the last session, participants were additionally asked if they had noticed any differences between the display types and whether they attended to the task. Finally, participants were debriefed and rewarded. Each session took approximately 1 h. All participants completed the four sessions within 2 weeks.

#### Data analysis

We separated discrimination sensitivity and response bias by applying a signal detection approach to the data (Macmillan & Creelman, 2005). The proportion of correct “diamond” reports to diamond targets was arbitrarily defined as the hit rate, and the proportion of false “diamond” reports to a “square” as the false alarm rate. Sensitivity *d′* and criterion *C* were computed separately for each mask stimulus and subsequently aggregated (Vorberg et al., 2003). Sensitivity values for the two mask shapes were then averaged. Criterion *C* values, however, were aggregated to a measure referred to as mask bias by the formula *C*_M_ = (*C*_square mask_ – *C*_diamond mask_)∕2. Values > 0 represent a tendency to respond accordingly to the mask, values < 0 a tendency to respond against it (Albrecht & Mattler, 2016).

We analyzed the effects of display type and polarity by computing separate 2 (display type) $\times$ 2 (polarity) $\times$ 7 (SOA) repeated-measures ANOVA with sensitivity *d′* and mask bias *C*_M_ as the respective dependent variable. All reported ANOVA *p* values in this study were Geisser–Greenhouse-corrected, but for readability, we state the uncorrected degrees of freedom. To assess interindividual variability as a function of display type and polarity, we divided participants into type-A and type-B observers based on visual inspection of the shape of their sensitivity function in the black-on-white polarity condition on CRT, which corresponds to our standard paradigm, and checked whether this pattern was consistent in the other conditions.

### Results

#### Signal detection analysis: sensitivity

**Fig. 6:**
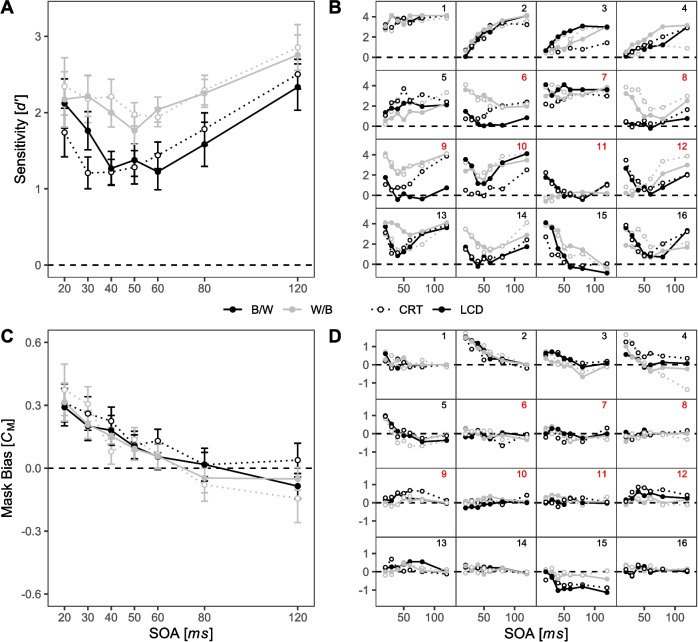
Signal detection measures across SOA, display type, and polarity conditions for Experiment 1. *Note.* Dotted lines represent sensitivity values from CRT, solid lines from LCD. Black lines and points depict sensitivity values of black-on-white polarity, gray lines and points depict white-on-black polarity. **A** Averaged masking functions. **B** Individual masking functions, sorted by visually assessed observer type. Numbers in the upper right corner of each panel are added for ease of reference. Panels 1–5 show plain type-A observers. Panels 6–10 show participants with ambiguous observer type. Panels 11-–16 show plain type-B observers. Red numbers indicate participants with inconsistent masking types across conditions. **C** Averaged mask biases; positive values reflect a tendency to respond according to the shape of the mask, negative values reflect a tendency to respond contrary to the shape of the mask. **D** Individual mask biases, sorted in the same way as in (**B**). Red numbers indicate participants with inconsistent masking types across conditions (see **B**). All error bars represent within-subject standard error of the mean following Loftus and Masson (1994)

Averaged sensitivity functions (Fig. 6A) reveal a substantial modulation of the masking effect across SOAs for all four display type × polarity conditions. A main effect of SOA, *F*(6, 90) = 4.18, *p* = 0.033, ${\eta }_{G}^{2}$ = 0.068, confirms this observation. Descriptively, all four averaged masking functions show a typical U-shaped curve. In addition, sensitivity was generally higher when stimuli were presented with a white-on-black polarity (*M* = 2.21, *SD* = 1.25) than with a black-on-white polarity (*M* = 1.63, *SD* = 1.29), as reflected by the significant main effect of polarity, *F*(1, 15) = 6.56, *p* = 0.022, ${\eta }_{G}^{2}$ = 0.054. Visual inspection of the averaged masking functions in Fig. 6A shows that higher sensitivities with white-on-black polarity were prevalent, especially with intermediate SOAs. In contrast, for the shortest SOA of 20 ms and the longest SOA of 120 ms, this difference between polarity conditions was negligible. The difference in average sensitivity between CRT (*M* = 1.93, *SD* = 1.25) and LCD (*M* = 1.92, *SD* = 1.35) was small, and neither the main effect of display type, *F*(1, 15) < 0.01, *p* = 0.954, nor the interaction display type × SOA, *F*(6, 90) = 2.35, *p* = 0.083, nor the interaction display type × polarity × SOA reached significance, *F*(6, 90) = 2.14, *p* = 0.111.

Visual inspection of Fig. 6A, however, suggested to us the possibility of a SOA-dependent reversion of relative masking strength between CRT and LCD displays for the black-on-white polarity condition: With short SOAs (20 and 30 ms), sensitivity was numerically higher on LCD (*M*_20ms_ = 2.12, *SD* = 1.43; *M*_30ms_ = 1.76, *SD* = 1.2) than on CRT (*M*_20ms_ = 1.74, *SD* = 1.22; *M*_30ms_ = 1.21, *SD* = 0.87), whereas with longer SOAs (80 and 120 ms), this effect was reversed, showing numerically higher sensitivities on CRT (*M*_80ms_ = 1.78, *SD* = 1.32; *M*_120ms_ = 2.5, *SD* = 1.1) than on LCDs (*M*_80ms_ = 1.58, *SD* = 1.61; *M*_120ms_ = 2.33, *SD* = 1.5). To examine this potential effect of display type in conditions with black-on-white stimuli, we conducted a separate exploratory ANOVA post hoc. The main effect of display type was not significant, *F*(1, 15) = 0.13, *p* = 0.723. However, the main effect of SOA, *F*(6, 90) = 4.97, *p* = 0.016*,* ${\eta }_{G}^{2}$ = 0.093, and the interaction display type × SOA reached significance, *F*(6, 90) = 3.12, *p* = 0.044, ${\eta }_{G}^{2}$= 0.013. According to this exploratory post hoc analysis, display type could lead to a shift of masking functions, as indicated in Fig. 6A.

Masking functions varied considerably among individuals and showed differences in the shape as well as the overall level of masking (Fig. 6B; cf. Albrecht et al., 2010; Albrecht & Mattler, 2012a, 2012b, 2016). Note that display type and polarity on the individual level are not well balanced across sessions due to the insufficient balancing procedure. Therefore, changes of masking functions between sessions may have resulted from condition-unspecific learning effects (for individual masking functions as function of session number, see Supplementary Figure S1).

Nevertheless, we would like to highlight three important points: (1) There is an intraindividual consistency of the shape of masking functions in nine participants, with five participants showing consistent type-A functions (Fig. 6B, participants 1–5) and four participants showing consistent type-B functions (participants 13–16). The remaining seven participants showed clear signs of masking, although the shape of masking functions varied substantially across sessions (participants 6–12). Interestingly, the shape of these participants’ masking function always changed between the second and the third session, and all participants showed type-B masking or close to ceiling performance (participant 7) in later sessions 3 and 4 (see Supplementary Figure S1). Within the first two sessions, only two participants changed from a type-B masking function in the LCD-B/W condition to a type-A function in the CRT-B/W condition (participants 6 and 9). We note that both of these participants were in the same balancing group. The remaining five participants showed type-A masking functions or near-guessing performance either in session 1 (participants 7, 10) or in sessions 1 and 2 (participants 8, 11, and 12).

(2) Higher sensitivities in one or both W/B polarity sessions occurred in seven participants (Fig. 6B, participants 4, 6, 8, 9, 13, 14, and 15). In five of them, both W/B polarity was presented in the final two sessions (participants 6, 8, 9, 13, and 14). Only one participant (participant 15) showed worse performance in the last session (LCD-B/W) than in the W/B conditions presented in the sessions before (see also Figure S1 ).

(3) The SOA-dependent reversion of relative masking strength between CRT and LCD that was apparent to us in the average masking functions of the B/W polarity condition in Fig. 6A is hardly found at the individual participant level. Only three participants showed a corresponding pattern of results with stronger masking for short and weaker masking for longer SOAs on CRT compared to LCD (Fig. 6B, participants 6, 9, and 15).

#### Signal detection analysis: Mask bias

Analysis of the mask bias *C*_M_ revealed a main effect of SOA (*F*(6, 90) = 5.90, *p* = 0.013, ${\eta }_{G}^{2}$ = 0.107, see Fig. 6C). Overall, the mask bias *C*_M_ decreases with increasing SOA, reflecting a tendency to respond in accordance with the mask for short but not for longer SOAs. This is a well-known effect which seems to be related to the use of percepts of apparent motion between target and mask stimulus as a response strategy. At short SOA, this motion is not produced by the stimulus sequence, which causes the strategy to induce a bias towards the mask shape (see, e.g., Albrecht & Mattler, 2012a). The effect did not differ between the four conditions indicated by nonsignificant effects of polarity and display type (all *F* ≤ 1.17, *p* ≥ 0.334). This observation is confirmed by the masking functions of the individuals in Fig. 6D. While some interindividual variability in the general course of *C*_M_ across SOA can be seen, for most participants, this pattern is highly consistent across display type and polarity conditions. In addition, a previously observed relationship between the shape of the masking function and the shape of the mask bias function is evident at the intraindividual level (Albrecht & Mattler, 2012a): Participants with type-A masking functions, who are known to rely on the use of apparent motion, tend to exhibit a stronger mask bias with short SOAs (cf. Fig. 6B and D, panels 1–5).

#### Phenomenological reports

**Fig. 7:**
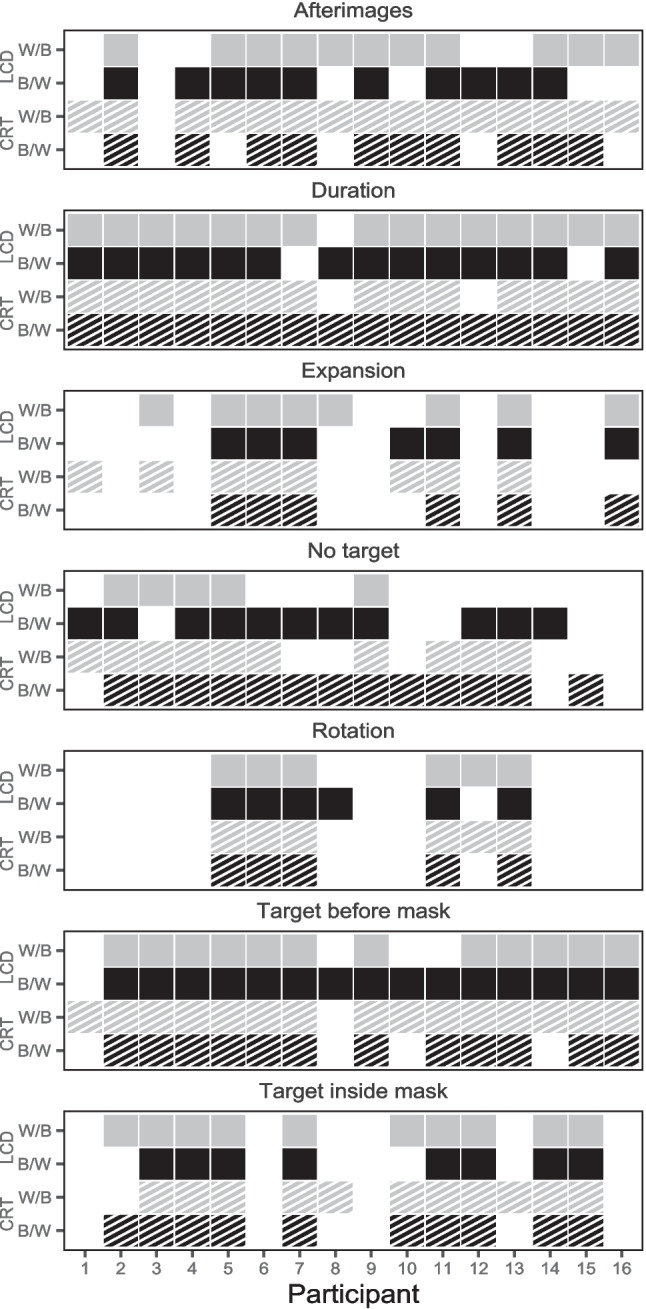
Phenomenological reports in Experiment 1. *Note.* Each panel depicts whether or not a participant responded affirmatively to a question regarding an experience of the corresponding type of percept in the current session. Affirmation of seeing a specific type of percept is marked by a square. The color and pattern of the square indicate the display type and polarity. Negation of a type of percept is depicted by a white square. Black squares represent confirmation of a type of percept in a black-on-white polarity session, and gray squares in a white-on-black polarity session. Squares with a striped pattern represent confirmation of a type of percept in a CRT session, and homogeneous squares in an LCD session

Results of the phenomenological questionnaire are presented in Fig. 7. Evaluation of the 16 participants’ answers to the seven phenomenological questions revealed 72 of 112 cases (64.3%) where participants reported seeing the same types of percepts across all display type and polarity conditions (in 51 cases, the same percept was reported in all four conditions; in 21 cases, none of the percepts were reported in all four conditions). Moreover, in 185 of 224 cases (82.6%), participants affirmed to experience the same type of percept irrespective of display type, indicating an inter-individually stable experience of percepts regardless of display type (χ^2^(1) = 85.1, *p* < 0.001). Similarly, in 173 cases (77.2%), participants affirmed to experience the same type of percept irrespective of polarity, indicating an inter-individually stable experience of percepts regardless of polarity (χ^2^(1) = 54.5, *p* < 0.001). On average, participants reported seeing *M* = 4.52 types of percepts per session. Neither display type nor polarity had a strong effect on the average number of reported types of percepts (*M*_CRT+B/W_ = 4.5, *M*_CRT+W/B_ = 4.94, *M*_LCD+B/W_ = 4.44, and *M*_LCD+W/B_ = 4.19).

Some percepts were generally reported more often than others. While the percepts *changes in perceived target duration*, *target before mask*, *no target*, and *afterimages* were reported by almost every participant in at least one session, the percepts *rotation* and *expansion* were reported less frequently. Although these results have to be interpreted with caution due to the small number of observations and the crude method of measurement by a questionnaire, the data suggests that the reports of the percepts that we examined here do not depend systematically on polarity and display type.

### Discussion

In Experiment 1 we observed typical metacontrast masking functions for all four combinations of display type and polarity. Interindividual variability of masking functions was high, and reports from a post-experimental questionnaire suggest that the phenomenology was not modulated by display type or polarity. These results replicate previous findings with CRTs (Albrecht & Mattler, 2012a, 2012b, 2016; Albrecht et al., 2010; Koster et al., 2020). In summary, metacontrast masking seems to be robust regarding display technology, as the basic patterns of metacontrast masking can be observed with either technology.

Larger masking effects were found with black-on-white rather than white-on-black polarity. Similar effects of stimulus-background polarity on metacontrast masking have been reported before (e.g., Breitmeyer et al., 2008; Breitmeyer, 1978b; Kolers, 1962; Sherrick et al., 1974). In these studies, masking tended to be weaker with white stimuli presented on a darker background than with black stimuli presented on a brighter background. Beside this overall effect, effects of contrast polarity seem to depend on different variables, like SOA, stimulus size, and criterion content (Breitmeyer et al., 2008; Breitmeyer, 1978b). However, it should be noted that most previous studies on contrast polarity effects in masking used black and white stimuli on a mid-gray background. In contrast, the present study used black stimuli on a white background and white stimuli on a black background. Therefore, reduced masking effects in the white-on-black condition in the present study might be caused by the overall increased background brightness in the black-on-white condition compared to the white-on-black condition.

Although statistically nonsignificant, the average masking functions with black-on-white polarity pointed to the possibility that display type might produce slightly different masking functions with black-on-white polarity after all. For short SOA (20 to 30 ms), masking was relatively stronger on LCD, while for long SOA (60 to 120 ms), masking was stronger on CRT. This possibility is in contrast to the fact that this inversion of masking is virtually absent in individuals’ data. However, this absence could be due to the balancing of polarity and display type across participants. To clarify the possibility of such an effect of display type, we replicated the black-on-white polarity condition in a follow-up experiment.

The effect of display type that has been reported by Kihara and colleagues (2010) could not be replicated in Experiment 1. These authors examined masking effects with green-on-black stimuli and found reduced masking effects on LCD for intermediate SOAs. However, the data in the corresponding white-on-black polarity condition of Experiment 1, which is based on more (16 rather than 12) participants and more (52 rather than 20) trials per condition than in the previous study, did not provide evidence for such an effect of display type.

## Experiment 2

Experiment 2 was designed to replicate the black-on-white polarity conditions of Experiment 1 where the averaged masking functions pointed to the possibility of a modulatory effect of display type, although this effect was not confirmed by statistical analyses. As mentioned above, the design of Experiment 1 could not rule out learning effects, because display type and session were not fully balanced across participants. In Experiment 2, we focused on black-on-white polarity, examined more participants, included a separate practice session, and carefully balanced the conditions to increase chances of detecting a potential effect of display type.

### Methods

#### Participants

A new sample of *N* = 24 students participated in Experiment 2 (aged between 18 and 32 years, *M* = 22.29, *SD* = 2.91; 17 female). The same exclusion criteria as in Experiment 1 applied. One participant had to be excluded because she stopped working properly on the task over the course of the experiment and was replaced by another participant. All participants gave informed written consent before attending the experiment and received either a monetary reward or course credit. All experiments were approved by the local ethics committee of the Georg-Elias-Müller-Institute of Psychology, University of Göttingen, and all experimental procedures are in accordance with the Declaration of Helsinki.

#### Sample size rationale

We estimated power and required sample size using the same procedures as in Experiment 1, except that we based the simulations on the effect size of display type × SOA interaction found in the follow-up analyses of Experiment 1. Simulations (*n* = 10,000) indicated a power of 79.7% (Greenhouse–Geisser-corrected) with a sample size of *n* = 24 participants. The number of trials per participant and condition was identical to Experiment 1, so that the precision at the individual level did not change in this respect. In addition, however, we have now also introduced a training session to reduce learning effects in the data, which in turn should lead to an increase in the expected individual precision. It can therefore be assumed that the actual statistical power of Experiment 2 is higher than the value we estimated with our simulations. Furthermore, the total number of observations per condition is higher than in Experiment 1 (24 participants × 104 observations per condition = 2496 observations).

#### Apparatus, stimuli, procedure, and data analysis

The same displays and setup as in Experiment 1 were used for stimulus presentation. Stimuli were the same, except that all stimuli were presented in the black-on-white polarity. The experiment consisted of three sessions. The first session was considered practice and run on either CRT or LCD. This new practice session was introduced to reduce learning effects in Experiment 2. The second and third sessions were the actual experimental sessions, and displays alternated between sessions. The four resulting orders of display type in the three sessions were balanced across participants. A total number of 2184 trials were run on each participant. In the questionnaire, we now followed Koster et al. (2020) more closely and distinguished the percepts *dark target* and *bright target* instead of putting them together into the percept *afterimages* as in Experiment 1. To keep the questionnaire short, the percept *changes of perceived target duration* was excluded, since it was almost always affirmed in Experiment 1 (in 59 of the 64 cases). The data from the two experimental sessions was analyzed, and the first block of each session was discarded as a practice block, leaving 48 for signal detection measure computation. A 2 (display type) $\times$ 7 (SOA) repeated-measures ANOVA was run separately for each signal detection measure.

### Results

#### Signal detection analysis: Sensitivity

**Fig. 8:**
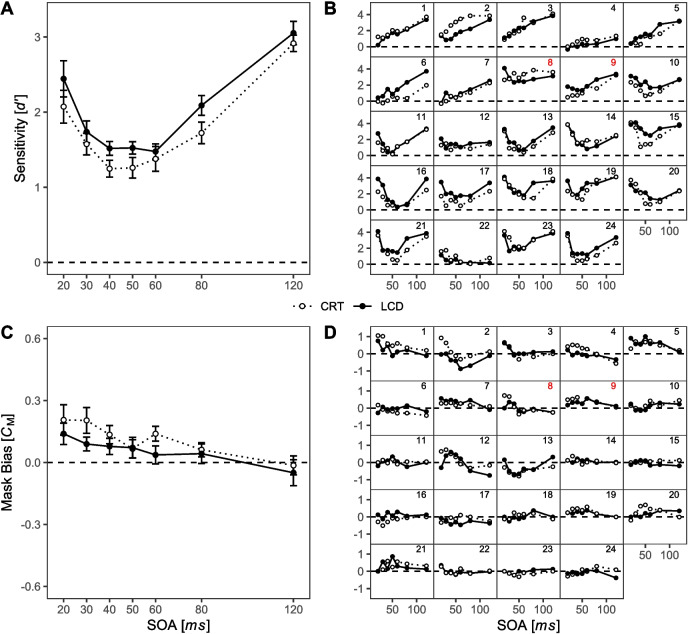
Signal detection measures across SOA and display type for Experiment 2. *Note.* Dotted lines represent sensitivity values from CRT, solid lines represent sensitivity measures from LCD. Stimuli were presented with the black-on-white polarity only. **A** Averaged masking functions. **B** Individual masking functions, sorted by visually assessed observer type. Numbers in the upper right corner of each panel are added for ease of reference. Panels 1–7 show plain type-A observers. Panels 8 and 9 show participants with ambiguous observer type. Panels 10–24 show plain type-B observers. Red numbers indicate participants with inconsistent masking types across conditions. **C** Averaged mask biases; positive values reflect a tendency to respond according to the mask shape, negative values reflect a tendency to respond contrary to the mask shape. **D** Individual mask biases, sorted in the same way as **B**. Red numbers indicate participants with inconsistent masking types across conditions. All error bars represent within-subject standard errors of the mean, following Loftus and Masson (1994)

Averaged sensitivity values are given in Fig. 8A. The main effect of SOA was significant, *F*(6, 138) = 19.45, *p* < 0.001, ${\eta }_{G}^{2}$ = 0.233, confirming the typical modulation of masking effects by SOA. Descriptively, we observed U-shaped masking functions for both display type conditions. The difference in average sensitivity between CRT (*M* = 1.45, *SD* = 1.14) and LCD (*M* = 1.76, *SD* = 1.1) was again small, but we observed a marginally significant main effect of display type, *F*(1, 23) = 4.24, *p* = 0.051, ${\eta }_{G}^{2}$ = 0.014, indicating better performance on LCD. Visual inspection of the masking functions shows that performance was higher on LCD at virtually every SOA. Most important, the interaction SOA $\times$ display type did not reach significance, *F*(6, 138) = 0.85, *p* = 0.484.

Visual inspection of the individual masking functions in Fig. 8B shows a substantial variability with *n* = 7 consistent type-A observers and *n* = 15 consistent type-B observers. The remaining *n* = 2 participants showed different masking functions with different display types, with type-A masking functions on CRT and a tendency to type-B masking functions on LCD. Notably, presentation order differed between these two participants (participant 8: session 2 on LCD and session 3 on CRT; participant 9: session 2 on CRT and session 3 on LCD; see also Supplementary Figure S2). A tendency for higher sensitivity on LCD than CRT, as implied by the marginal main effect of display type described above, can also be observed on an individual level, although only one participant shows this effect for all SOAs (participant 17). Individual masking functions for each session are given in Supplementary Figure S2.

#### Signal detection analysis: Mask bias

Averaged mask bias values are given in Fig. 8C. The main effect of SOA on mask bias was marginally significant, *F*(6, 138) = 2.55, *p* = 0.066, ${\eta }_{G}^{2}$ = 0.037, with a higher bias towards the mask for short SOAs, decreasing with increasing SOA. Neither the main effect of display type, *F*(1, 23) = 2.19, *p* = 0.153, nor the interaction SOA $\times$ display type was significant, *F*(6, 138) = 0.74, *p* = 0.554. On an individual level, participants with type-A masking functions tend to show a more pronounced mask bias at short SOA (see Fig. 8D, participants 1–5, 8 on CRT devices).

#### Phenomenological reports

**Fig. 9:**
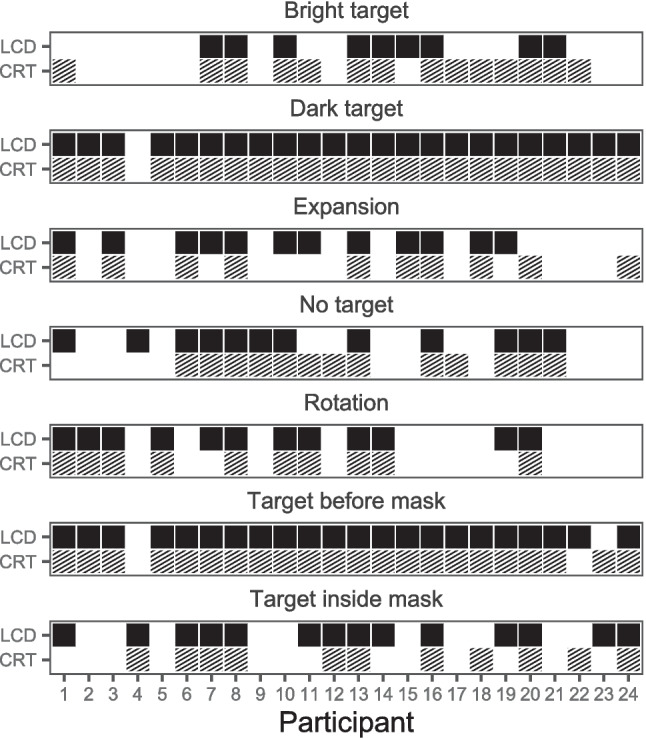
Phenomenological reports in Experiment 2. *Note.* Each panel depicts self-reported availability of one of the seven types of percept for each of the *N* = 24 participants. Confirmation of having seen a type of percept is marked by a square of the respective conditions pattern, negation by leaving that area white. Black squares with a striped pattern represent confirmation of a percept in a CRT session and pattern-less squares in an LCD session. The polarity was black-on-white for both display types

An overview of the results of the individual perceptual reports can be found in Fig. 9. Evaluation of the 24 participants’ answers to the seven phenomenological questions revealed 139 of 168 cases (82,7%) where the reported types of percepts did not differ between display type (χ^2^_(1)_ = 65.1, p < 0.001). On average, participants affirmed *M* = 4.31 types of percepts per session, which was comparable across display types (4.29 with CRT, 4.33 with LCD). The percepts *target before mask* and *dark target* were affirmed by almost every participant and most of the time in both sessions. The other types of percepts were affirmed with lower frequencies and questions regarding *expansion* and *rotation* received the fewest affirmations. Effects of display type on perceptual reports, assessed by comparison of absolute report frequencies, seem negligible. The largest differences occurred for *target inside mask* and *bright target*. To sum up, the present data suggests that phenomenological reports are not substantially altered by display types.

## Analysis of a unified sample

**Fig. 10:**
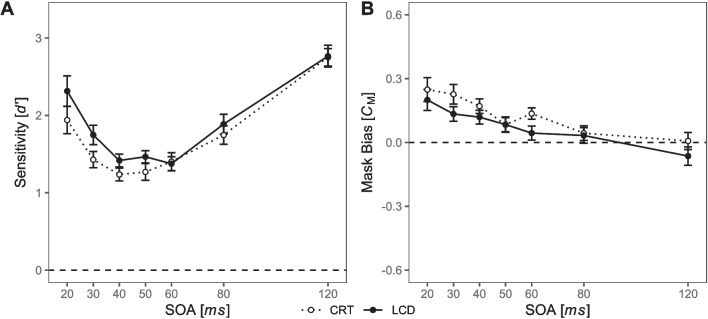
Signal detection measures across SOA and display type for the unified sample. *Note.* Dotted lines represent sensitivity values from CRT, solid lines from LCD. Only data for the B/W condition are considered here. **A** Averaged masking functions. **B** Averaged mask biases; positive values reflect a tendency to respond according to the mask shape, negative values reflect a tendency to respond contrary to the mask shape. All error bars represent within-subject standard error of the mean, following Loftus and Masson (1994)

Visual inspection of the average masking functions of Experiment 1 pointed to the possibility of an interaction SOA × display type in the black-on-white polarity condition, but this effect was not confirmed by the data of Experiment 2. However, visual inspection of the average masking functions of the two experiments reveals that masking effects were increased with the CRT compared to the LCD at the two shortest SOAs of 20 ms and 30 ms in both experiments (compare Figs. 6A and 7A). Since the individual experimental sessions were identical in both experiments, data from the two sessions with the black-on-white polarity condition of Experiment 1 and the two experimental sessions of Experiment 2 were merged into one dataset and analyzed post hoc with repeated-measures ANOVAs with independent variables display type and SOA, and dependent variables *d*′ and mask bias. Averages of measures of sensitivity and bias are displayed in Fig. 10.

The main effect of SOA was significant on *d*′, *F*(6, 234) = 22.75, *p* < 0.001, ${\eta }_{G}^{2}$ =. 163. However, neither the main effect of display type, *F*(1, 39) = 2.73, *p* = 0.106, nor the interaction SOA $\times$ display type reached significance, *F*(6, 234) = 2.16, *p* = 0.086 (see Fig. 10A).

Regarding mask bias, the main effect of SOA was significant, *F*(6, 243) = 6.31, *p* < 0.001, ${\eta }_{G}^{2}$ = 0.05. The main effect of display type failed to reach significance, *F*(1, 39) = 3.28, *p* = 0.078, and the interaction SOA $\times$ display type was not significant, *F*(6, 243) = 0.94, *p* = 0.446.

## General discussion

In two experiments, we compared metacontrast masking on displays with CRT and LCD technologies. Experiment 1 additionally compared masking effects across two stimulus-background polarity conditions. Regardless of display type, typical metacontrast masking effects were observed in both experiments with masking functions that varied substantially between individuals, and participants reported a rich subjective phenomenology. Visual inspection of the averaged masking functions of Experiment 1 pointed to the possibility of an interaction of display type and SOA in the black-on-white polarity condition. To examine this possibility, we ran Experiment 2 as a replication of the black-on-white polarity condition of Experiment 1, but it revealed no evidence for this interaction. Moreover, the marginally significant main effect of display type that occurred in Experiment 2 was not confirmed when the corresponding data of Experiment 1 and Experiment 2 were analyzed together. Beyond this, Experiment 1 revealed a significant main effect of polarity on metacontrast masking, indicating that black-on-white stimuli lead to more masking than white-on-black stimuli, and this effect was not modulated by display type or SOA. Regarding mask bias, we found evidence for a bias to respond in accordance with the mask that decreased with increasing SOA, which accords well with previous findings (cf. Albrecht & Mattler, 2012a, 2016; Albrecht et al., 2010). This mask bias effect was neither modulated by stimulus polarity nor by display type. These findings suggest that the investigation of metacontrast masking with LCD will not generate other phenomena than those that have been reported in the literature on the basis of data that were gathered with CRT monitors.

The change in display devices has prompted a number of studies to examine whether visual phenomena that have been extensively studied with CRTs are modified by the use of LCDs. While several phenomena have proven to be robust to changing display types, the experiment by Kihara et al. found that changing display types changes metacontrast masking (2010). In an attempt to replicate this finding, we used an improved design to clarify whether metacontrast masking is actually modified by display types. Although our results accord with the main conclusion of Kihara et al. (2010) that basic metacontrast masking effects can be obtained with LCD, we could not replicate the effect of display type described in their study. Kihara and colleagues reported an interaction of display type and SOA with stronger masking at intermediate SOA of 33 and 55 ms on CRT. The failure of replication is most evident when comparing the experimental conditions of Kihara et al. with the corresponding conditions of the present study, which are the white-on-black polarity conditions in Experiment 1, where no display effects occurred at all.

On the one hand, one may argue that this failure of replication is due to differences between the two studies. First, Kihara et al. presented their stimuli at 60 Hz, while we ran our displays at 100 Hz. This difference could play a role, if one follows Kihara et al., who speculated that increased masking effects with CRT as compared to LCD may result from the greater instantaneous peak of luminance of the mask on CRT as compared to LCD. With 100 Hz, the total luminance of a stimulus is distributed over more peaks of luminance per time interval on the CRT than with 60 Hz, leading to relatively lower peaks with 100 Hz. Therefore, the difference between the instantaneous peak of luminance of the CRT and the luminance signal of the LCD might be reduced in the present study as compared to the study of Kihara and colleagues. We note, however, that this view of Kihara et al. is at odds with a view that refers to Bloch's law (Gorea, 2015) assuming that the effects of duration and intensity can be accounted for jointly by their product, yielding stimulus energy, which is a commonly accepted variable in the metacontrast masking literature (e.g., Breitmeyer & Öğmen, 2006). Following this established view, in order to equalize stimulus energy, the luminance of the stimuli has been matched between CRT and LCD displays in the present study as well as in the previous study. We note, however, that the view of Kihara and colleagues predicts that metacontrast masking is modulated by the refresh rate on CRTs but not LCDs, since the maximum of the instantaneous luminance signal is not modulated by refresh rate for LCDs. This could be tested in future experiments.

A second potentially important difference between experiments concerns the stimuli that were used. Kihara and colleagues presented target and mask stimuli with no spatial gap in between, while the triangular area which constituted the missing corner of the target amounted to 0.08° of visual angle height between target and mask. The authors speculated that the blurring that occurs in CRT displays may have reduced the size of the missing corner and thus increased the masking effects on the CRT compared to LCD, where this blurring effect does not occur. In the present study, the four empty triangular areas that occur when a target is placed inside the star-shaped cutout of the mask had a height of 0.26° visual angle each (see Fig. 5A). Thus, if blur on the CRT does indeed affect masking, this effect may be greater in the Kihara et al. study than in the present study because the larger empty triangular areas of the stimulus material in the present study should be less susceptible to blur effects than the small missing corners in the stimulus material of Kihara and colleagues. As a result, there would be less of a difference between display types in the present study. On the other hand, in the present study, the target and the mask were separated by a continuous gap of 0.02° visual angle around the target (Fig. 5A). It is reasonable to assume that the blur on the CRT may have reduced this continuous gap between the target and the mask, an effect that cannot occur in the stimuli of Kihara and colleagues. Following this reasoning, the masking effect on the CRT would be enhanced in the present study due to the blur-induced reduction in target-mask distance, which in turn would predict larger display effects in the present study compared with the study by Kihara and colleagues.

Finally, one may argue that the present study failed to replicate the display type effect reported by Kihara et al. because of the unexpectedly high overall performance of our participants in the white-on-black polarity condition of Experiment 1. Accompanying ceiling effects could have occluded the display type effect with SOAs of 33 and 50 ms as reported by Kihara et al. in our data. We note, however, that the comparison of performance levels between the two studies is complicated by the fact that we used a forced-choice task with two alternatives, whereas Kihara and colleagues used a forced-choice task with four alternatives, reducing chance performance to 25%. Irrespective of this, however, in Experiment 1, masking at short SOAs was numerically greater with the LCD rather than the CRT (Fig. 6A), while Kihara et al. found greater masking with the CRT (see their Fig. 4).

It is not clear whether any of these differences between studies accounts for the failure of replication of the display-type effect of Kihara and colleagues' (2010), especially since each of the above points refers to relatively small differences. Overall, therefore, it seems unlikely that there are any real effects of display type on metacontrast masking, even under circumstances consistent with the specific conditions used by Kihara and colleagues (e.g., 60 Hz, green stimuli on a black background, no gap between target and mask, etc.). It seems much more likely that the factors we were able to control for in the present study are responsible for the results of Kihara et al. These include the sophisticated matching procedure we used to achieve comparable settings with both display types, including background luminance and ambiance. Another important factor is based on the significant individual differences in both perceptual learning and metacontrast masking functions. This is demonstrated by the data in the present study, which are consistent with a large body of evidence in the literature (e.g., Albrecht & Mattler, 2012b, 2016; Albrecht et al., 2010). When these individual data are aggregated across participants, differences between conditions may arise, leading to erroneous conclusions. To counteract such artifacts, it is important to study a sufficiently large sample of participants. It is also important to increase the reliability of measurements by training participants sufficiently for the task and giving them sufficient rest periods between blocks of trials. Therefore, we studied large samples of participants in several smaller sessions spread over several days, and collected a relatively large number of measurement replicates for each of our experimental conditions. Since our experiments were set up in the same way as previous experiments from research on metacontrast, we believe that our results can be considered valid evidence for the view that metacontrast masking does not differ between CRT and LCD screens. This conclusion corresponds with other research comparing various visual phenomena between CRT and LCD technologies where strong effects of display technology seem to be rare (see Introduction). To sum up, the present results encourage the use of LCDs for the study of metacontrast masking because comparable effects can be obtained when display properties like refresh rate, display resolution, luminance, and color are carefully aligned. On the other hand, however, the findings challenge theories of metacontrast masking by demonstrating that the physical differences in the stimulus associated with the two display technologies do not modulate metacontrast masking. Given that these fundamental physical differences between displays evoke different neural responses on early levels of visual processing up to V1 (Gawne & Woods, 2003; Herbst et al., 2013; Krolak-Salmon et al., 2003; Williams, 2004; Zele & Vingrys, 2005), the absence of display type effects challenge theories of metacontrast masking which locate essential mechanisms at early levels of processing in the visual hierarchy. The present results rather suggest that metacontrast masking is based on mechanism that are located at processing levels after V1.

Finally, the present findings suggest that beyond the effects of stimulus polarities, differences between studies in metacontrast masking are more likely caused by interindividual differences between participants and perceptual learning effects rather than by differences in display technologies. Thus, rather than the technology of the displays, the present findings point to individual differences that should be taken into account in studies on metacontrast masking (Albrecht & Mattler, 2012b, 2016).

## Conclusion

We compared metacontrast masking between two fundamentally different types of display technologies, a traditional CRT and a more recent LCD. Despite the fundamental physical differences between the two devices, we carefully matched the displays to achieve a high degree of agreement in several physical and perceptual parameters. We obtained strong masking effects with both displays. In addition, more specific aspects of the phenomenon of metacontrast masking, including interindividual variability, rich phenomenology, and specific response bias patterns, were also consistent. Results support current theoretical accounts of metacontrast masking that are based on mechanisms that are not modulated by the fundamental differences between these display technologies. The present results place metacontrast masking among the set of perceptual phenomena that have been shown to be robust to the change in display types from CRT to LCD, and contribute to the view that the inevitable switch to LCD displays will not impede progress in vision research.

## Supplementary Information

Below is the link to the electronic supplementary material.

Supplementary file1 (PDF 74 KB)

Supplementary file2 (PDF 84 KB)

Supplementary file3 (PDF 235 KB)

Supplementary file4 (PDF 248 KB)

## Data Availability

The data and analysis code for all reported experiments is available in a public project on the Open Science Framework: https://osf.io/vcztx None of the studies have been preregistered.

## Footnotes

1 Note that the response time of our LCD display for a transition from black to white was 5.90 ms, while the response time for white to black was 1 ms. Kihara and colleagues reported these values for their LCD display only in the form of their Fig. 1C. We estimated their response times using https://apps.automeris.io/wpd/ and found values of 5.82 and 1.11 ms for the transition from black to white and from white to black, respectively.

## Code availability

See above.
