# Supplementary material for: Metacontrast masking does not change with different display technologies: A comparison of CRT and LCD monitors
Source: Behav Res Methods. 2024 Dec 30;57(1):30. doi: 10.3758/s13428-024-02526-w (PMC11685275; doi:10.3758/s13428-024-02526-w)
Supplement: Supplementary file 1 — Supplementary file1 (PDF 74 KB) [file 13428_2024_2526_MOESM1_ESM.pdf]

Supplementary Material

S1 Method for measuring the luminance signal

We wanted to measure not only one simply transition per display type and polarity condition but wanted to approximate the luminance signal course of full experimental trials i.e., the presentation of a target and mask and the transition between them, since here the critical effects were expected.

Since target and mask are not presented at the same location in our paradigm, we were not able to measure both stimuli at once and had to separate the measurements. To align the two measurements afterwards in time, a reference stimulus was presented at the beginning of each measurement. This reference stimulus consisted of a single black to white shift, regardless of polarity condition. Following the reference stimulus, the background color of the respective polarity condition was presented for 480 ms. When measuring a target, the placeholder stimulus was then presented for a duration of 20 ms. Mask measurements followed the same scheme, but presentation was delayed by the respective SOA and the placeholder was presented for a duration of 120 ms. In the following post processing, we first determined the start of the reference stimulus for each measurement by applying a rolling window maximum search and computed the overall maximum and minimum of the measurement as well as the standard deviation. We then defined a series of local maxima larger than the overall maximum - 1 SD as a phase of white stimulation and series of local maxima smaller than the overall minimum + 1 SD as a phase of black stimulation. The amplitude value after the first shift from black to white was then defined as the beginning of the reference stimulus. Subsequent the timeline was centered on the time point of planned target presentation by adding 500 ms onto the beginning of reference

presentation. For plotting, amplitude values of a measurement were normalized to values between 0 and 1. Note that absolute amplitude differences between these measurements are meaningless, since we adjusted the iris of the OTR-3 measurement device to obtain a maximum amplitude of approx. 2 V to avoid signal artefacts of too low or high signal. All measurements were conducted with the photodiode mounted on a small tripod and in close distance to the display surface.
