# Supplementary material for: Metacontrast masking does not change with different display technologies: A comparison of CRT and LCD monitors
Source: Behav Res Methods. 2024 Dec 30;57(1):30. doi: 10.3758/s13428-024-02526-w (PMC11685275; doi:10.3758/s13428-024-02526-w)
Supplement: Supplementary file 2 — Supplementary file2 (PDF 84 KB) [file 13428_2024_2526_MOESM2_ESM.pdf]

Supplementary Material

S2 Method for luminance and color matching

Measurements were made with the measurement devices touching the display surface in an otherwise completely dark room. Prior to measurements the displays were warmed up for at least 30 min. Ideally, we would have wanted to measure luminance and color of the actual stimuli, but because they were smaller than the sensor sizes of the measurement devices, we were forced to use a larger placeholder stimulus instead. Target and mask were therefore approximated by a 5.5 × 5.5 cm square, placed in the center of the screen. This stimulus diameter was 197 % larger than the target stimulus and 71 % larger than the mask. Polarity conditions were considered as well, so that either a black or white placeholder was presented upon a background of the opposite color. Measurements were taken from five different locations on the screen: One from the center of the stimulus placeholder and four from the background area adjacent to each side of the square. Since background luminance and color varied considerably between measurement locations, the average was computed and used for comparison in the matching process.

In order to be comparable to previous research (e.g., Albrecht & Mattler, 2010, 2012, 2016) we intended a luminance of 74.5 cd/m² for white and 0.37 cd/m^2 for black. Note that the typical luminance of black stimuli in our previous studies on metacontrast was lower and the luminance for white stimuli higher (namely 0.03 and 72.3 cd/m^2 respectivley, e.g., Albrecht & Mattler, 2010, 2012b, 2016). However, the rather small contrast range of the LCD required a tradeoff so that we couldn't obtain such a low value for black together with a sufficiently high value for white and exact matching of the white value was hard to achieve in the present study

because we tried to matched both, luminance and color between the two types of displays at the same time.

The matching process itself was compromised of two major steps. First, both displays white point was set to approx. 6500K and minimal and maximal luminance was coarsely adjusted to the intended levels through displays contrast and brightness settings. Second, this coarse match was refined by creating separate *ICC profiles* for each of the four display and polarity combinations. Therefore, we iteratively adjusted minimal and maximal values of the three basic color channels until luminance and color of all white and black areas across display and polarity conditions were matched a closely as possible. Final measurements were repeated 10 times and averaged to validate.

Following the displays luminance and color calibration spectral profiles and ambient luminance were assessed for each display and polarity combination. For the spectral profile measurement, displays were filled with each of the three basic colors and 10 measurements per condition were taken in the displays center. Since spectral profiles did not vary much between polarity conditions, we averaged across polarity. Ambient luminance was measured from the headrest at 1m distance, pointing straight towards the display and using a diffusor head. Since sensor size was not a problem for these measurements, we presented a target stimulus instead of the placeholder at the center of the screen. Stimulus and background were presented in the colors of the respective polarity condition.

References

Albrecht, T., & Mattler, U. (2010). Individual differences in metacontrast masking: A call for caution when interpreting group data. *Consciousness and Cognition*, *19*(2), 672–673. https://doi.org/10.1016/j.concog.2010.03.010

Albrecht, T., & Mattler, U. (2012). Individual differences in metacontrast masking regarding sensitivity and response bias. *Consciousness and Cognition*, *21*(3), 1222–1231. https://doi.org/10.1016/j.concog.2012.04.006

Albrecht, T., & Mattler, U. (2016). Individually different weighting of multiple processes underlies effects of metacontrast masking. *Consciousness and Cognition*, *42*, 162–180. https://doi.org/10.1016/j.concog.2016.03.006
