# Supplementary material for: Metacontrast masking does not change with different display technologies: A comparison of CRT and LCD monitors
Source: Behav Res Methods. 2024 Dec 30;57(1):30. doi: 10.3758/s13428-024-02526-w (PMC11685275; doi:10.3758/s13428-024-02526-w)
Supplement: Supplementary file 3 — Supplementary file3 (PDF 235 KB) [file 13428_2024_2526_MOESM3_ESM.pdf]

**Supplementary Figure**

**S1 Individual masking functions by session for Experiment 1**

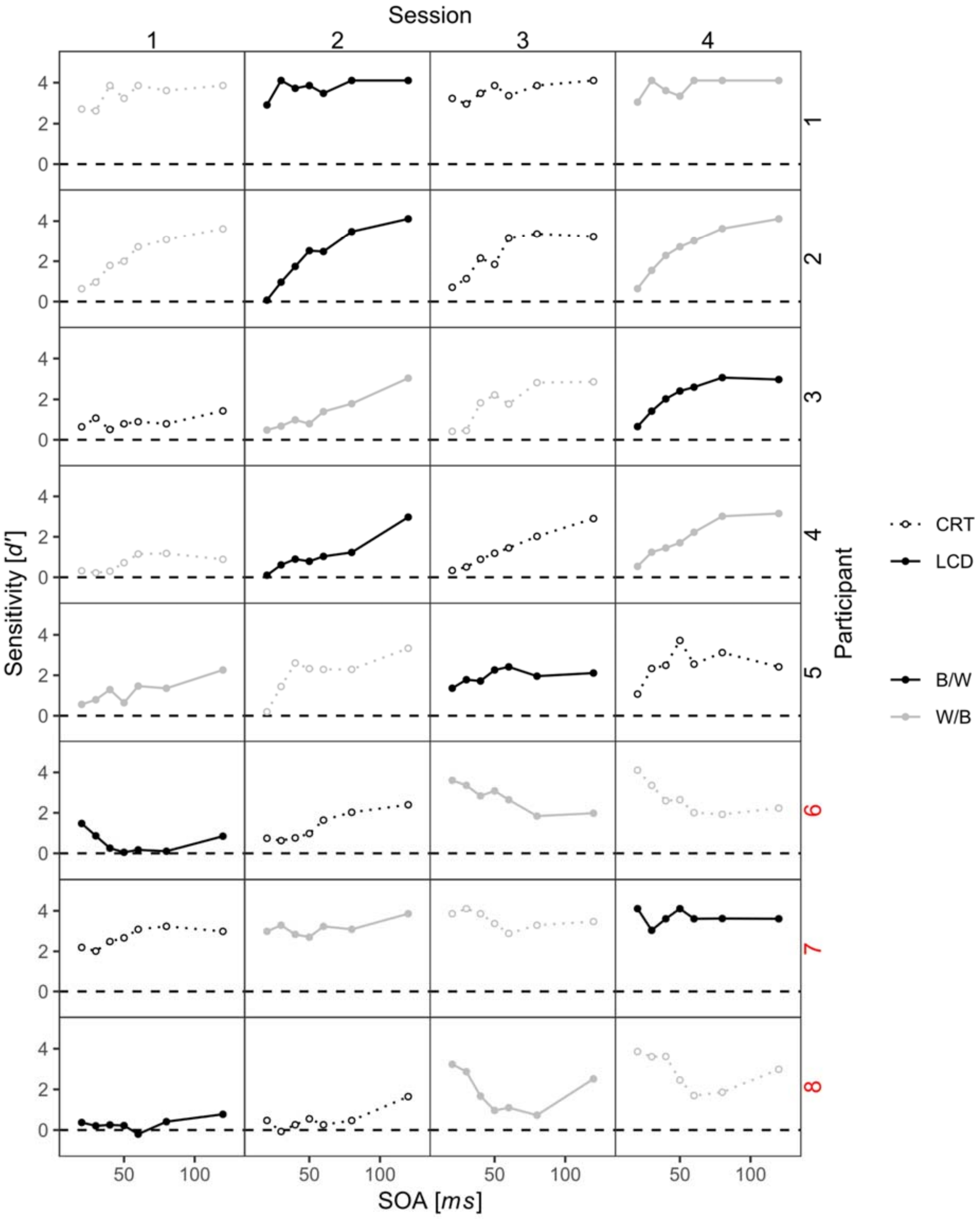

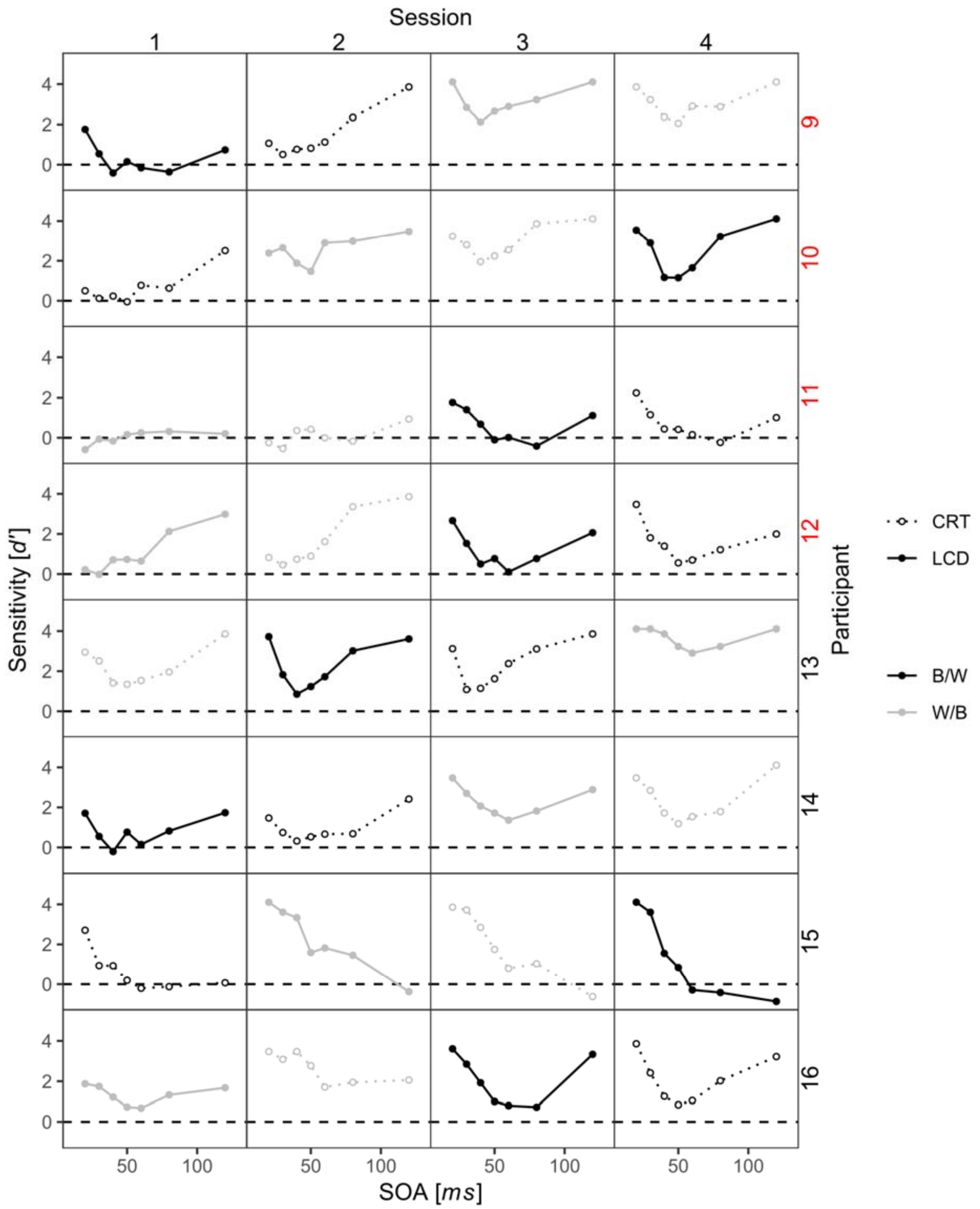


*Figure S1*. Individual masking functions from Experiment 1, sorted by visually assessed observer type and session number. Numbers in the upper right corner of each panel are for ease of reference and represent the number of experiment (1), participant number (1-16) and session

number (1-4) for ease of reference. Red numbers indicate participants with inconsistent masking types across conditions.
