# Supplementary material for: Metacontrast masking does not change with different display technologies: A comparison of CRT and LCD monitors
Source: Behav Res Methods. 2024 Dec 30;57(1):30. doi: 10.3758/s13428-024-02526-w (PMC11685275; doi:10.3758/s13428-024-02526-w)
Supplement: Supplementary file 4 — Supplementary file4 (PDF 248 KB) [file 13428_2024_2526_MOESM4_ESM.pdf]

Supplementary Figure

S2 Individual masking functions by session for Experiment 2

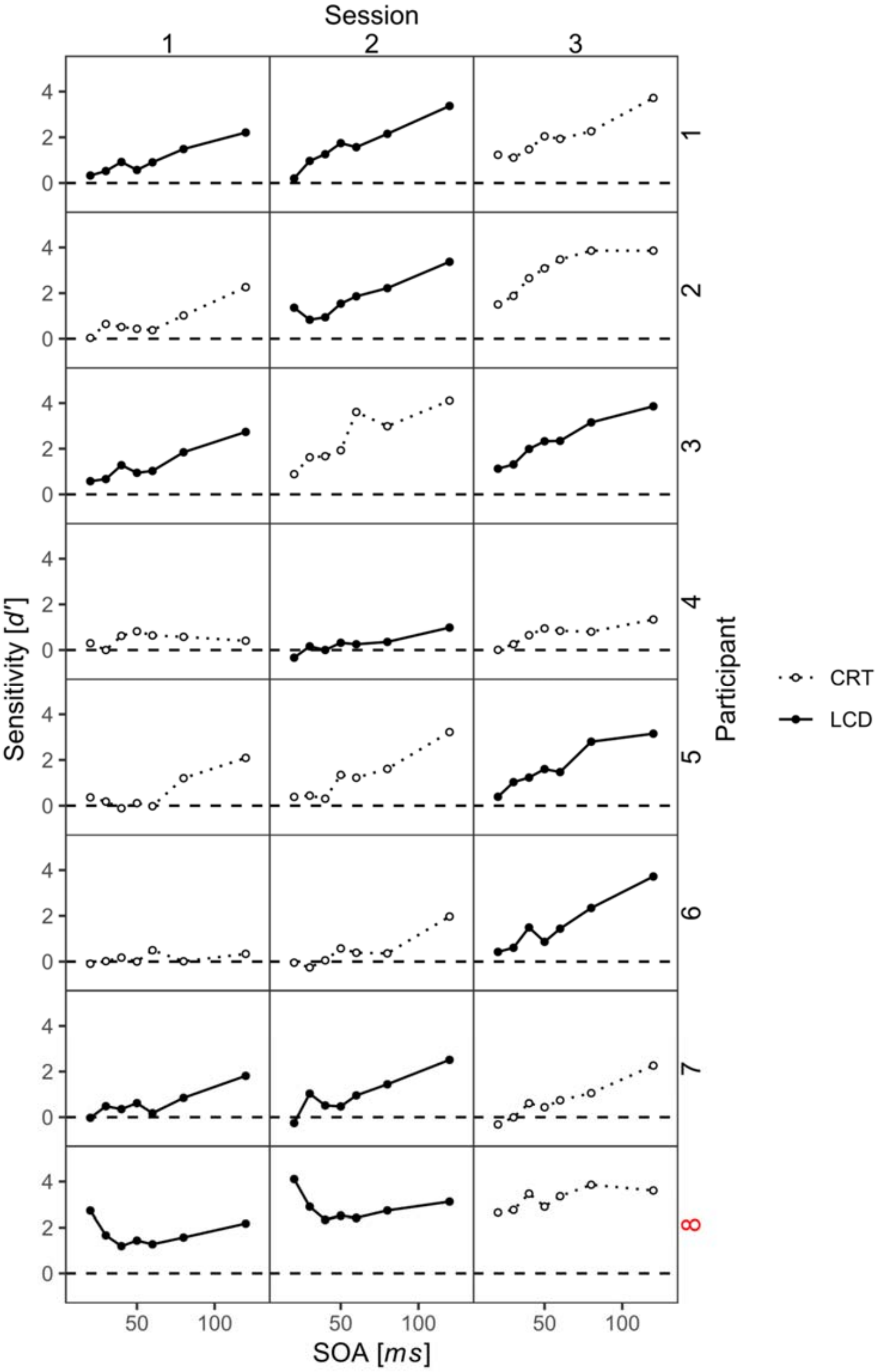

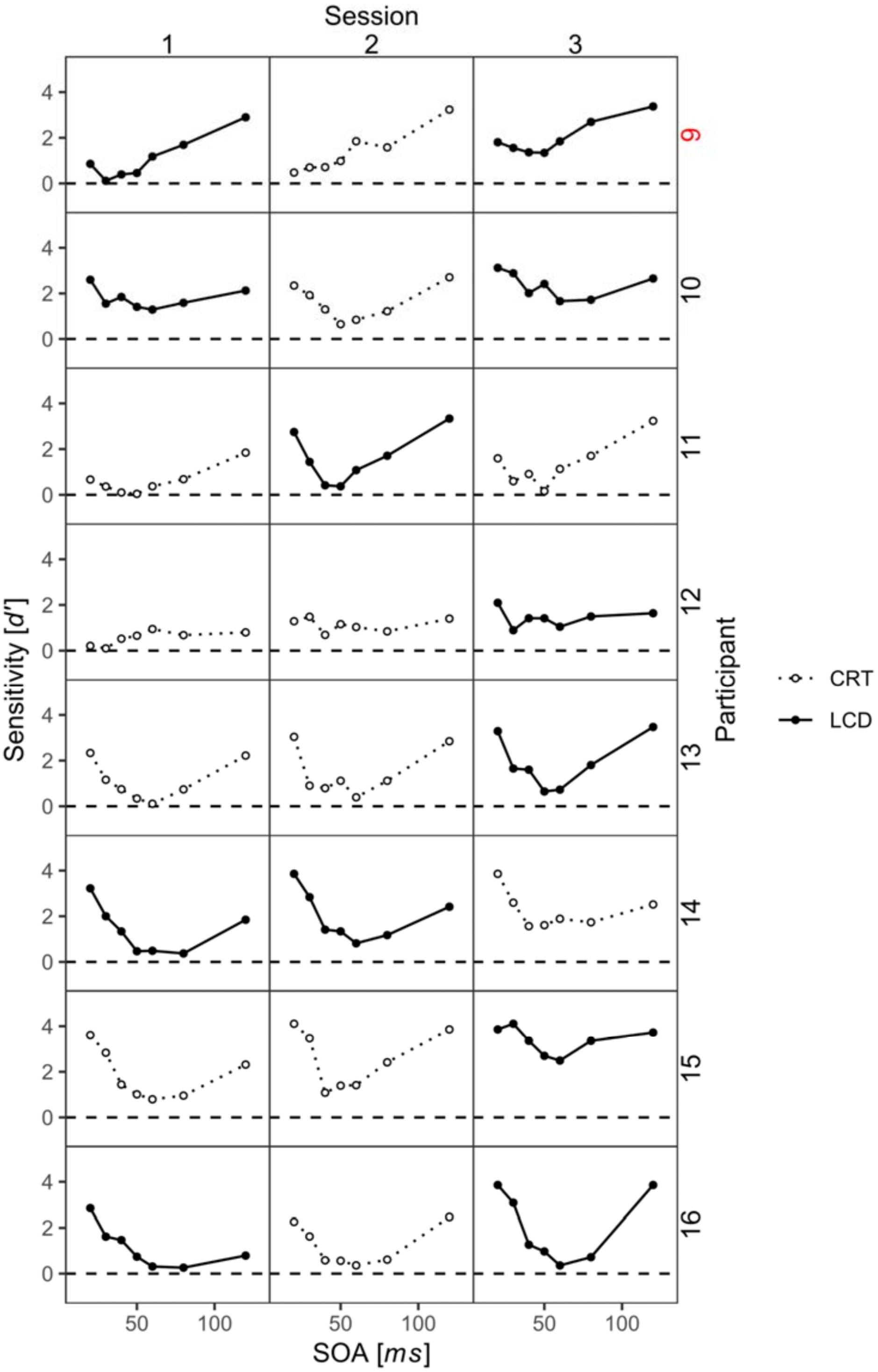
Session
1
2
3
Sensitivity [d']
Participant
9
10
11
12
13
14
15
16
SOA [ms]
50
100
0
2
4
CRT
LCD

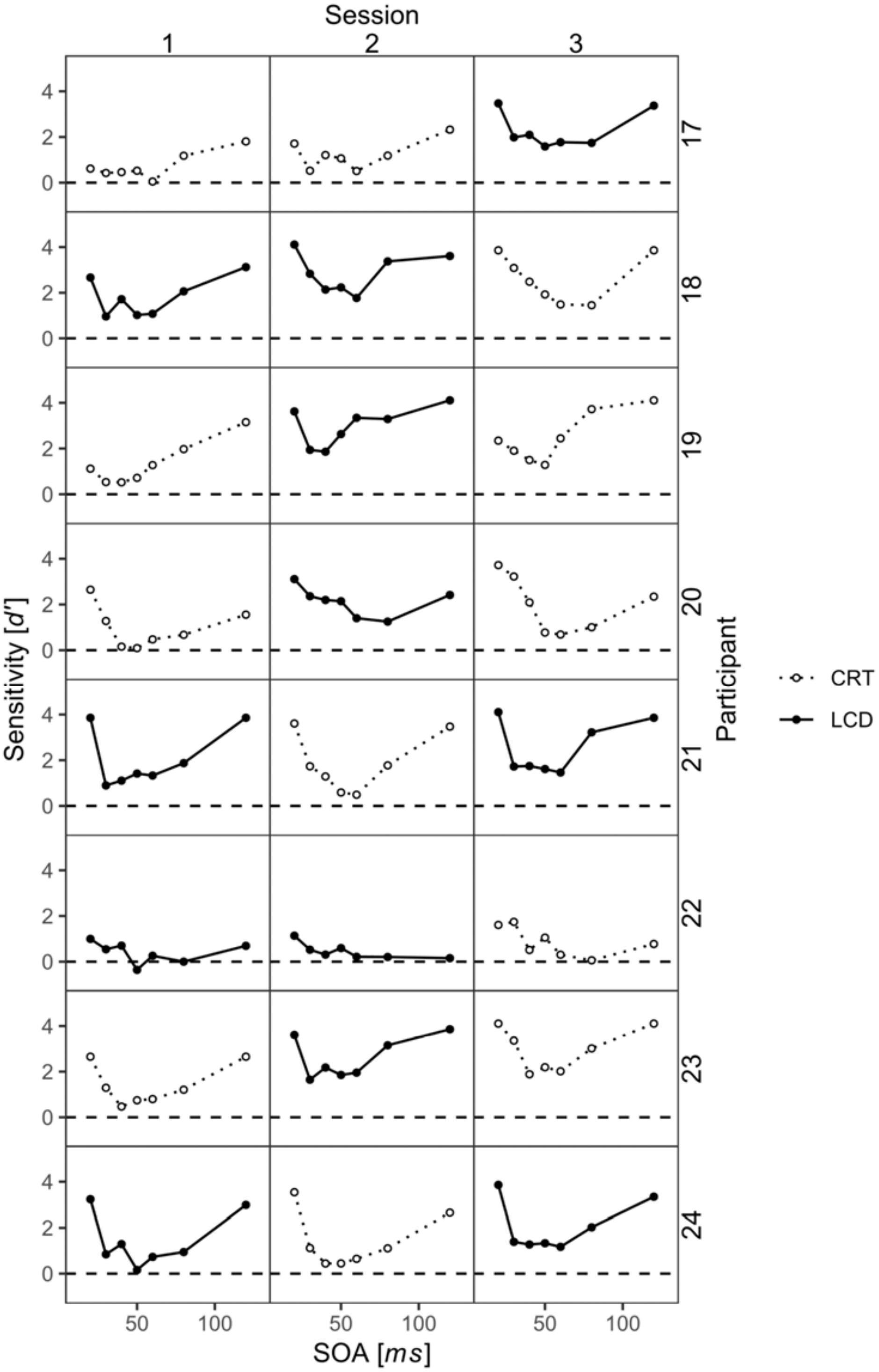

Figure S2. Individual masking functions from Experiment 2, sorted by visually assessed observer type and session number. Stimulus polarity condition was always black-on-white. Numbers in the upper right corner of each panel are for ease of reference and represent the number of experiment (2), participant number (1-24) and session number (1-3) for ease of reference. Red

numbers indicate participants with inconsistent masking types across conditions. Note that the first session in Experiment 2 was considered a training session and was therefore discarded from all further analysis.
